# Reactivation of Latent HIV-1 Expression by Engineered TALE Transcription Factors

**DOI:** 10.1371/journal.pone.0150037

**Published:** 2016-03-02

**Authors:** Pedro Perdigão, Thomas Gaj, Mariana Santa-Marta, Carlos F. Barbas, Joao Goncalves

**Affiliations:** 1 Research Institute for Medicines (iMed ULisboa), Faculdadede Farmácia, Universidade de Lisboa, Lisboa, Portugal; 2 The Skaggs Institute for Chemical Biology, The Scripps Research Institute, La Jolla, California, United States of America; 3 Departments of Chemistry, The Scripps Research Institute, La Jolla, California, United States of America; 4 Department of Cell and Molecular Biology, The Scripps Research Institute, La Jolla, California, United States of America; Osaka University, JAPAN

## Abstract

The presence of replication-competent HIV-1 –which resides mainly in resting CD4^+^ T cells–is a major hurdle to its eradication. While pharmacological approaches have been useful for inducing the expression of this latent population of virus, they have been unable to purge HIV-1 from all its reservoirs. Additionally, many of these strategies have been associated with adverse effects, underscoring the need for alternative approaches capable of reactivating viral expression. Here we show that engineered transcriptional modulators based on customizable transcription activator-like effector (TALE) proteins can induce gene expression from the HIV-1 long terminal repeat promoter, and that combinations of TALE transcription factors can synergistically reactivate latent viral expression in cell line models of HIV-1 latency. We further show that complementing TALE transcription factors with Vorinostat, a histone deacetylase inhibitor, enhances HIV-1 expression in latency models. Collectively, these findings demonstrate that TALE transcription factors are a potentially effective alternative to current pharmacological routes for reactivating latent virus and that combining synthetic transcriptional activators with histone deacetylase inhibitors could lead to the development of improved therapies for latent HIV-1 infection.

## Introduction

Over the past two decades, numerous advances in the treatment of HIV/AIDS have significantly increased the lifespan–and quality of life–of individuals infected with HIV type 1 (HIV-1). Highly active antiretroviral therapy (HAART), in particular, has emerged as a powerful treatment option, capable of decreasing plasma viral loads to below the limit of detection of many clinical assays [1–3]. Yet despite its effectiveness, HAART does not cure patients of HIV-1 infection, due to the existence of residual latent and replication-competent virus hidden in cellular reservoirs [4–8]. This population of cells, which consists mainly in resting memory CD4^+^ T cells, harbors integrated proviral DNA that re-emerges shortly after discontinuation of HAART. HIV-1 latency is typically established when activated CD4^+^ T cells become infected with the virus and revert back to a resting memory state [8]. These cells are thus non-permissive for viral gene expression and refractory to many treatments, including HAART. Although the mechanisms behind latency are complex [8,9], they likely involve: (*i*) the absence of key host transcription factors that drive transcriptional initiation [10,11] or elongation [12,13] in resting CD4^+^ T cells; (*ii*) low levels of the trans-activator of transcription (Tat) regulatory protein [14]; (*iii*) proviral integration into condensed chromatin regions [15,16] or expressed regions that become silenced by promoter occlusion [17–20]; and (*iv*) the induction of epigenetic modifications that can inhibit viral gene expression, including DNA methylation [21,22] and histone deacetylation [23].

Because the presence of latent HIV-1 represents an enormous barrier toward its eradication, numerous strategies have been developed to purge it from its cellular reservoirs. Chief among these has been activation of latently infected T cells via treatment with cytokines [24] or monoclonal antibodies [25], as well as NF-κB stimulation via protein kinase C agonists [26,27]. Histone deacetylase (HDAC) inhibitors, such as valproic acid [28] and Vorinostat [29], have also proven capable of inducing viral gene expression by disrupting recruitment of HDAC proteins to the HIV long terminal repeat (LTR) promoter [30,31]. These approaches, however, have been unable to eradicate virus from all latent pools and have even been associated with adverse effects, including severe immune reactions [32–35]. As a result, new strategies capable of inducing viral gene expression are needed to enable the development of next-generation HIV-1 therapeutics.

The emergence of customizable DNA-binding platforms, including engineered zinc-finger [36] and transcription activator-like effector (TALE) [37] proteins, as well as CRISPR-Cas9 [38], has provided investigators with a set of tools capable of sequence-specific DNA recognition [39]. TALE proteins, in particular, have now been utilized to create a broad range of tools capable of gene modification and regulation, including transcriptional activators [40,41] and repressors [42], nucleases [40,43,44], site-specific recombinases [45] and epigenetic effectors [46–48]. The DNA binding domain of a TALE protein consists of a series of repeat domains, each ~34 amino acid residues in length, that coordinate to recognize a single base pair (bp) via two adjacent amino acid residues, termed repeat variable diresidues (RVDs) [49,50]. A variety of approaches have now been developed that enable rapid construction of custom TALE arrays capable of recognizing nearly any contiguous sequence [51,52]. As a result, TALEs have achieved widespread use throughout biotechnology, with the potential to impact future developments in human gene therapy.

Numerous studies have also demonstrated the utility of genome engineering for combating HIV/AIDs. Specifically, zinc-finger based transcriptional repressors [53–56], in addition to RNA interference [57–59], have proven effective at inhibiting HIV replication. Targeted nucleases have also demonstrated the capacity to excise integrated proviral DNA from infected cells [60–62] and confer HIV resistance to cells by inducing knockout of the primary co-receptors for HIV infection [44,63–65]. Targeted gene regulation technologies may also prove effective at reversing HIV-1 latency. Specifically, due to their versatility and ability to stimulate robust levels of gene expression in a highly specific manner [66], TALE transcription factors [40] could be used to stimulate viral gene expression within latent HIV-1 reservoirs, providing new means for enabling “shock and kill” therapy. Here we demonstrate that TALE transcription factors can be engineered to recognize the HIV-1 LTR promoter and induce viral gene expression in cell line models of HIV-1 latency. We also show that complementing TALE transcription factors with HDAC inhibitors can further enhance TALE-induced activation of latent HIV-1 expression. These findings indicate that TALE transcription factors are potentially effective tools for reactivating latent virus and could contribute to the development of next-generation HIV-1 therapies.

## Materials and Methods

### Plasmid construction

The HIV-1 pNL4-3 plasmid [67] was obtained from the NIH AIDS Research and Reference Reagent Program. pTat expression plasmid was kindly provided by Dr. Maryanne Simurda (State University of New York, Buffalo). TALEs were generated as previously described [51,68] using the Golden Gate TALEN and TAL Effector Kit 2.0 (Addgene ID: 1000000024) [52]. TALE arrays were cloned into the BsmBI restriction site of pcDNANT-T-VP64 [69] to generate pTLT-1 through 10. The pTALE-TF reporter vectors were constructed through PCR by amplifying the luciferase gene from pGL3-Basic (Promega, Madison, WI, USA) using the primers 5’ TALE-Luc-TLT1 through 10, which contained four direct repeats of each TALE binding site and 3’ Luc-Rev. PCR products were digested and cloned into the XhoI and SphI restriction sites of pGL3-Basic to generate pGL3-TLT-1 through 10. The HIV-1 LTR reporter plasmid was constructed by PCR amplifying the U3-R region of the LTR promoter from pNL4-3 using the primers 5’ LTR-Fwd and 3’ LTR-Rev. PCR product was digested and cloned into the XhoI and SphI restriction sites of pGL3-Basic to generate pGL3-LTR. Correct construction of each plasmid was verified by sequence analysis (S1 Table). Primer sequences are provided in S2 Table.

### Cell culture

Human embryonic kidney 293T (HEK293T) (American Type Culture Collection; ATCC) cells were cultured in Dulbecco’s modified Eagle’s medium (DMEM; Life Technologies, Carlsbad, CA, USA) supplemented with 10% (v/v) fetal bovine serum (FBS; Gibco, Carlsbad, CA, USA), 2 mM L-glutamine and 1% (v/v) antibiotic-antimycotic (Anti-Anti; Gibco, Carlsbad, CA, USA). J-Lat clones (NIH AIDS Reagents) were cultured in RPMI-1640 medium (Life Technologies, Carlsbad, CA, USA) supplemented with 10% (v/v) FBS, 2 mM L-glutamine and 1% (v/v) Anti-Anti. Cells were maintained at 37°C in a humidified atmosphere of 5% CO_2_.

### Luciferase assays

Luciferase assays were performed as previously described [68]. Briefly, HEK293T cells were seeded onto 96-well plates at a density of 4 x 10^4^ cells per well. At 16–24 h after seeding, cells were transfected with 200 ng of pTLT-1 through pTLT-10, 5 ng of pGL3-TLT-1 through pGL3-TLT -10 and 1 ng of pRL-CMV (Promega, Madison, WI, USA) using Lipofectamine 2000 (Life Technologies, Carlsbad, CA, USA) according to the manufacturer’s instructions. At 48 h after transfection, cells were washed once with Dulbecco’s PBS (DPBS; Life Technologies, Carlsbad, CA, USA) and lysed with Passive Lysis Buffer (Promega, Madison, WI, USA). Luciferase expression was measured with the Dual-Luciferase Reporter Assay System (Promega, Madison, WI, USA) using a Veritas Microplate Luminometer (Turner Biosystems, Sunnyvale, CA, USA) according to the manufacturer’s instructions. Normalized luciferase activity was determined by dividing firefly luciferase activity by Renilla luciferase activity.

### Western blots

HEK293T cells were seeded onto a 6-well plate at a density of 5 x 10^4^ cells per well. At 16–24 h after seeding, cells were transfected with 5 μg of pTLT-1 through pTLT-10 or pcDNA backbone vector by the calcium phosphate method [70]. At 48 h after transfection, cells were harvested and lysed with RIPA buffer (25 mM Tris-HCl, 150 mM NaCl, 1% NP-40, 1% sodium deoxycholate and 0.1% SDS, pH 7.6) supplemented with EDTA-free Protease Inhibitor Cocktail Tablets (Roche, Basel, Switzerland). The Bio-Rad Protein Assay Kit (Bio-Rad, Hercules, CA, USA) was used to determine protein concentration according to the manufacturer’s instructions. TALE transcription factor expression was analyzed by 4–12% SDS-PAGE (National Diagnostics, Atlanta, GA, USA). Samples were transferred onto a 0.2 μm nitrocellulose membrane as described [71] and detected with Immobilon Western Chemiluminescent HRP substrate (Millipore, Billerica, MA, USA) and Amersham HyperfilmECL (GE Healthcare, Little Chalfont, UK) chemiluminescence film. TALE transcription factors were detected by horseradish peroxidase-conjugated anti-HA monoclonal antibody (Roche, Basel, Switzerland). β-actin was used as an internal control and detected using a mouse anti-β-actin monoclonal antibody (Sigma, St. Louis, MO, USA) and horseradish peroxidase-conjugated goat anti-mouse IgG antibody (Bio-Rad, Hercules, CA, USA) (kindly provided by Dr. Cecília Rodrigues).

### HIV-1 reactivation

J-Lat cells were seeded onto a 10 cm dish at a density of 1 x 10^5^ cells per mL. At 48 h after seeding, 2 x 10^5^ cells per transfection were centrifuged at 100 x g for 10 min at room temperature and resuspended in Nucleofector Solution SE (Lonza, Basel, Switzerland) with 2 μg of pTLT-1 through pTLT-10 or pTat. Cells were transferred to 16-well Nucleocuvette Strips (Lonza, Basel, Switzerland) and electroporated by a 4D-Nucleofector System (Lonza, Basel, Switzerland) using the program CL-120, according to the manufacturer’s instructions. J-Lat cells were either left untreated or incubated with 10 ng/μL of TNF-α (R&D Systems, Minneapolis, MN, Canada). At 48 h after transfection, cells were washed twice with DPBS (Life Technologies, Carlsbad, CA, USA) and GFP expression was evaluated by flow cytometry (BD LSR II Flow Cytometer System; BD Biosciences, Franklin Lakes, NJ, USA). For each sample, 10,000 live events were collected, and data was analyzed using FlowJo (Tree Star, Inc., San Carlos, CA, USA).

### HDAC inhibitor treatments

J-Lat cells were seeded onto a 10 cm dish at a density of 2 x 10^5^ cells per mL. At 48 h after seeding, 1 x 10^6^ cells per transfection were centrifuged at 100 x g for 10 min at room temperature and resuspended in Nucleofector Solution V (Lonza, Basel, Switzerland) with 4 μg of pTLT-5 through pTLT-8. Cells were transferred to a Nucleocuvette (Lonza, Basel, Switzerland) and electroporated with an Amaxa Nucleofector II Device (Lonza, Basel, Switzerland) using the program X-001 according to the manufacturer’s instructions. At 24 h after transfection, J-Lat cells were treated with DMSO 0.1% or 330 nM, 660 nM or 1 μM of SAHA (NIH AIDS Reagents) [72] for 24 h. After treatment, cells were washed twice with DPBS and GFP expression was evaluated by flow cytometry analysis (BD LSR II Flow Cytometer System; BD Biosciences, Franklin Lakes, NJ, USA). For each sample, 10,000 live events were collected, and data was analyzed using FlowJo (Tree Star, Inc., San Carlos, CA, USA).

### Statistical analysis

Statistical analyses for all experiments were performed from three independent experimental replicates (*n* = 3) unless otherwise indicated. Two-tailed Student’s *t*-test was used for paired and unpaired samples (Prism Software 5.0, GraphPad Software).

## Results

### Designing TALE transcription factors to target the HIV-1 promoter

**Fig 1:**
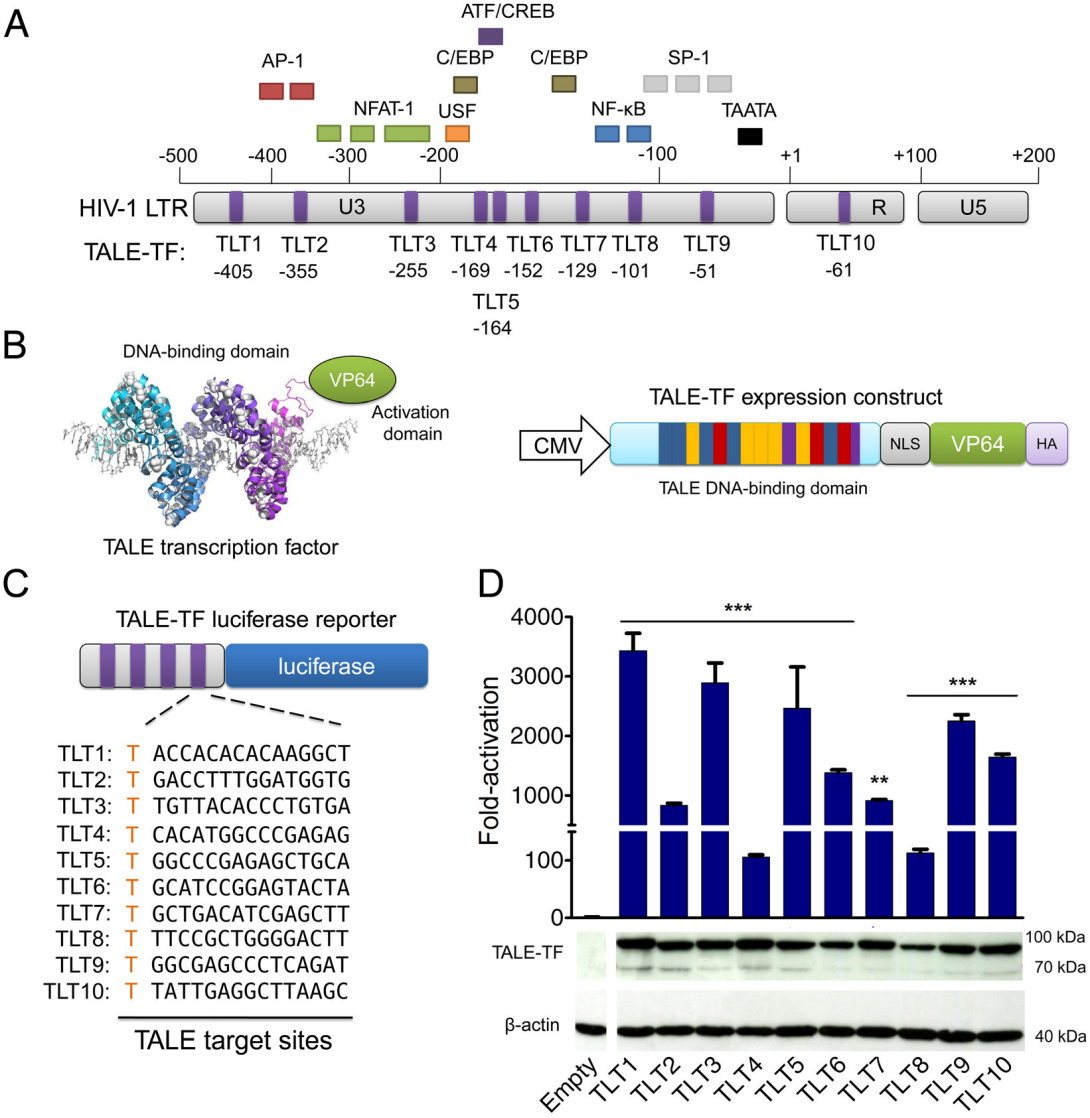
TALE transcription factors (TALE-TFs) designed to target the HIV-1 LTR promoter. **(A)** Schematic representation of the TALE transcription activator (TLT) binding sites within the HIV-1 long terminal repeat (LTR) promoter relative to the transcriptional start site (TSS) and main endogenous transcription factor binding sites. **(B) (Left)** Cartoon illustrating the structure of a TALE-TF, adapted from [109]. TALE repeats are colored cyan and purple, DNA shown as grey sticks. **(Right)** Schematic representation of the TLT expression construct used in this study. CMV indicates the cytomegalovirus promoter, TALE repeats are shown as individual bars (16 repeats total), VP64 denotes the tetrameric repeat of the herpes simplex virus VP16 transactivation domain, NLS stands for the nuclear localization signal derived from the simian virus (SV40) and HA indicates the hemagglutinin A tag. **(C)** Schematic representation of the luciferase reporter system containing four direct repeats of the TALE target sites for each TALE activator. Each TALE target site is shown. **(D) (Top)** Fold-activation of luciferase expression after co-transfection of TALE-TFs with luciferase reporter plasmid into HEK293T cells. Luciferase expression was normalized to cells transfected with reporter plasmid only. *Renilla* luciferase expression was used to normalize for transfection efficiency and cell number. Error bars indicate standard deviation of one experiment with three transfection replicates (*n* = 3; *p < 0.05; **p < 0.01; ***p < 0.001; *t*-test sample vs control (4x TALE binding site vector only)). (**Bottom)** Western blot of lysate from HEK293T cells transfected with TALE-TFs. Samples were taken 48 h after transfection and probed with horseradish peroxidase-conjugated anti-HA and anti-β-actin (loading control) antibodies. Empty indicates lysate from HEK293T cells transfected with empty pcDNA vector only.

We sought to reverse HIV-1 latency by inducing viral gene expression using engineered TALE transcription factors. We constructed ten TALE proteins designed to recognize distinct 16-bp sites within the HIV-1 LTR, the region of the virus that serves as its promoter (Fig 1A). TALE binding sites were constrained only by the presence of a 5’ thymidine (T_0_) nucleotide [69]. We fused each synthetic TALE array to VP64 [73], a tetrameric repeat of the herpes simplex virus VP16 transactivation domain, to generate synthetic transcriptional activators. VP64 is a widely used transactivation domain [47,74–79] capable of recruiting host cellular transcription factors to targeted genomic loci [80,81], but does not activate gene expression alone [73]. Each TALE transcription factor contained a C-terminal hemagglutinin (HA) tag and an internal nuclear localization signal (NLS) sequence between the DNA binding and transactivation domains (Fig 1A). The amino acid sequence of each protein is presented in S1 Table.

In order to determine whether each TALE could recognize its intended target site and induce gene expression, we adapted a previously described transient reporter assay [68] that correlates TALE-induced gene activation with increased luciferase expression. We inserted four direct repeats of each LTR binding site upstream of a luciferase reporter gene and co-transfected human embryonic kidney (HEK) 293T cells with reporter plasmid and expression vectors for each TALE activator (Fig 1C and 1D). This strategy was undertaken in order to increase reporter gene expression and more accurately evaluate TALE activity. Eight of the ten TALE activators (all but TLT4 and TLT8) induced a >800-fold increase in luciferase expression, with TLT1 (~3,400-fold), TLT3 (~2,900-fold), TLT7 (~2,500-fold) and TLT9 (~2,200-fold) inducing the highest levels of activation (*p* < 0.001) (Fig 1D). TLT4 and TLT8 achieved similarly high levels of absolute luciferase activity, but induced a modest ~100-fold increase in activation over mock-transfected cells. Even in the absence of a TALE activator, transfection of the TLT4 and TLT8 reporter plasmids led to a significant increase in luciferase expression (*p* < 0.001) (data not shown). Not surprisingly, however, the binding sites for TLT4 and TLT8 overlap with those recognized by the endogenous transcription factors C/EBP and NF-κB [82] (Fig 1A), respectively, indicating that native proteins could have been contributing to reporter gene activation. Compared to reporter plasmid only though, increased luciferase expression was evident after co-transfection with the specific TALE activator, indicating that TALEs have the potential to outcompete endogenous transcription factors for LTR binding sites.

Western blot analysis of HEK293T lysates also revealed that each TALE activator was expressed (Fig 1D). Low levels of a non-specific band (~70 KDa), however, were detected in several samples, possibly due to translation of a second open-reading frame present within the TALE mRNA transcript or recombination within the TALE DNA-binding domain, a phenomenon that can occur within a highly repetitive motif [83].

### TALE transcription factors activate gene expression from the HIV LTR

We next set out to test the ability of each TALE activator to stimulate transcription from the full-length U3 and R regions of the HIV-1 LTR using an episomal reporter assay. The U3-R regions of the LTR, in particular, contain the core promoter, enhancer and modulatory region, and regulate viral expression. Notably, unlike the transient reporter assay described above, which asked whether each TALE protein could bind its intended DNA target, this analysis aimed to evaluate the ability of each TALE activator to stimulate transcription from the full-length HIV-1 promoter.

**Fig 2:**
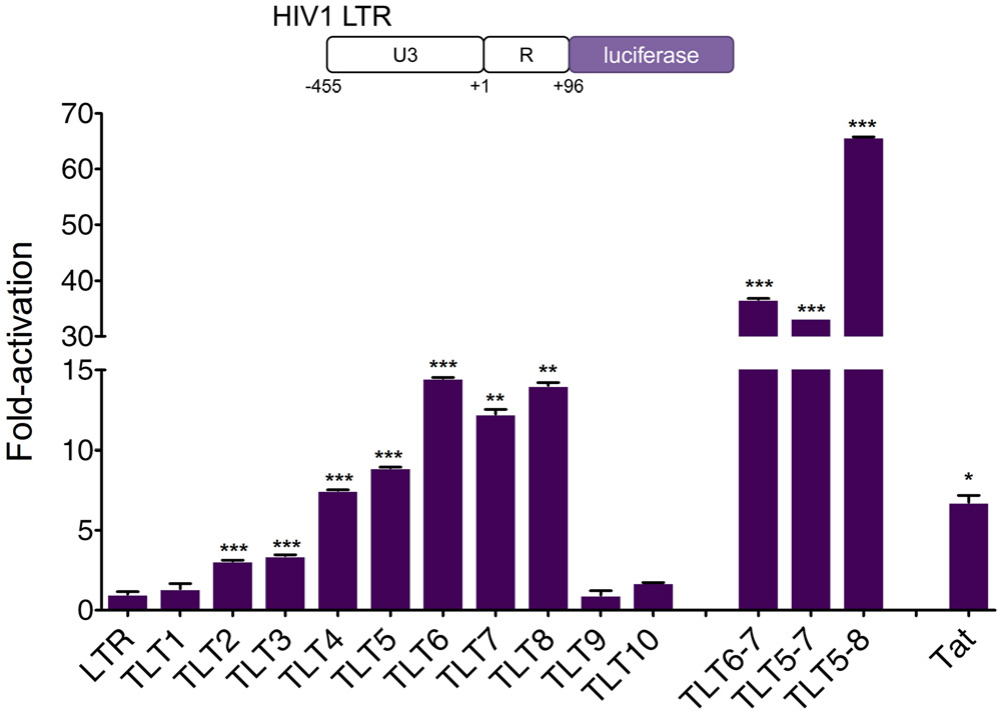
TALE-TF-mediated gene activation from the HIV-1 LTR promoter. **(Top)** Schematic representation of the luciferase reporter system used to evaluate TALE-TF activity from the HIV-1 LTR promoter. The U3 and R regions of the HIV LTR were placed upstream of the luciferase reporter. **(Bottom)** Fold-activation of luciferase expression in HEK293T cells co-transfected with reporter plasmid and TALE-TF or Tat expression vectors. Luciferase expression was normalized to cells transfected with reporter plasmid only. Error bars indicate standard deviation of one experiment with three transfection replicates (*n* = 3; *p < 0.05; **p < 0.01; ***p < 0.001; *t*-test sample vs. control (TLTNT)).

HEK293T cells were co-transfected with TALE activator and a reporter vector that contained the sequence between -455 and +96 from the LTR transcriptional start site (TSS) upstream of a luciferase reporter gene (Fig 2). We separately co-transfected an expression vector encoding the HIV-1 Tat protein as a positive control. Multiple activators, including TLT4, 5, 6, 7 and 8, induced a 7.5- to 14-fold increase in luciferase activity (*p* < 0.01), while Tat yielded only a ~7-fold increase in activation (Fig 2), likely because it stimulates transcriptional elongation more efficiently than initiation [84].

Previous reports have demonstrated that co-delivery of combinations of TALE transcription factors can lead to a synergistic increase in gene expression via cooperative effects that could mimic those associated with natural transcriptional processes [77,78]. The most potent TALE activators, TLT5, 6, 7 and 8, were designed to recognize a small region of the LTR between -170 and -100 bp from the TSS (Fig 1A). Indeed, sequence analysis of different strains of HIV-1 subtype B (i.e. the most predominant subtype across Europe, America, Australia and Japan) revealed that the binding sites for these TALEs are generally well conserved (S3 Table). We thus co-transfected HEK293T cells with LTR reporter plasmid and different combinations of TALE activators to test whether these proteins could be used in tandem to further enhance gene expression. Increased gene activation was observed for each set tested, most notably with a ~70-fold increase in luciferase expression after co-transfection with TLT5, 6, 7 and 8 (hereafter referred to as TLT5-8) (*p* < 0.001) (Fig 2). Overall, these data demonstrate that TALE transcription factors designed to target the U3 and R regions of the HIV LTR promoter can induce efficient gene activation.

### Reactivation of latent HIV-1 by TALE transcription factors

**Fig 3:**
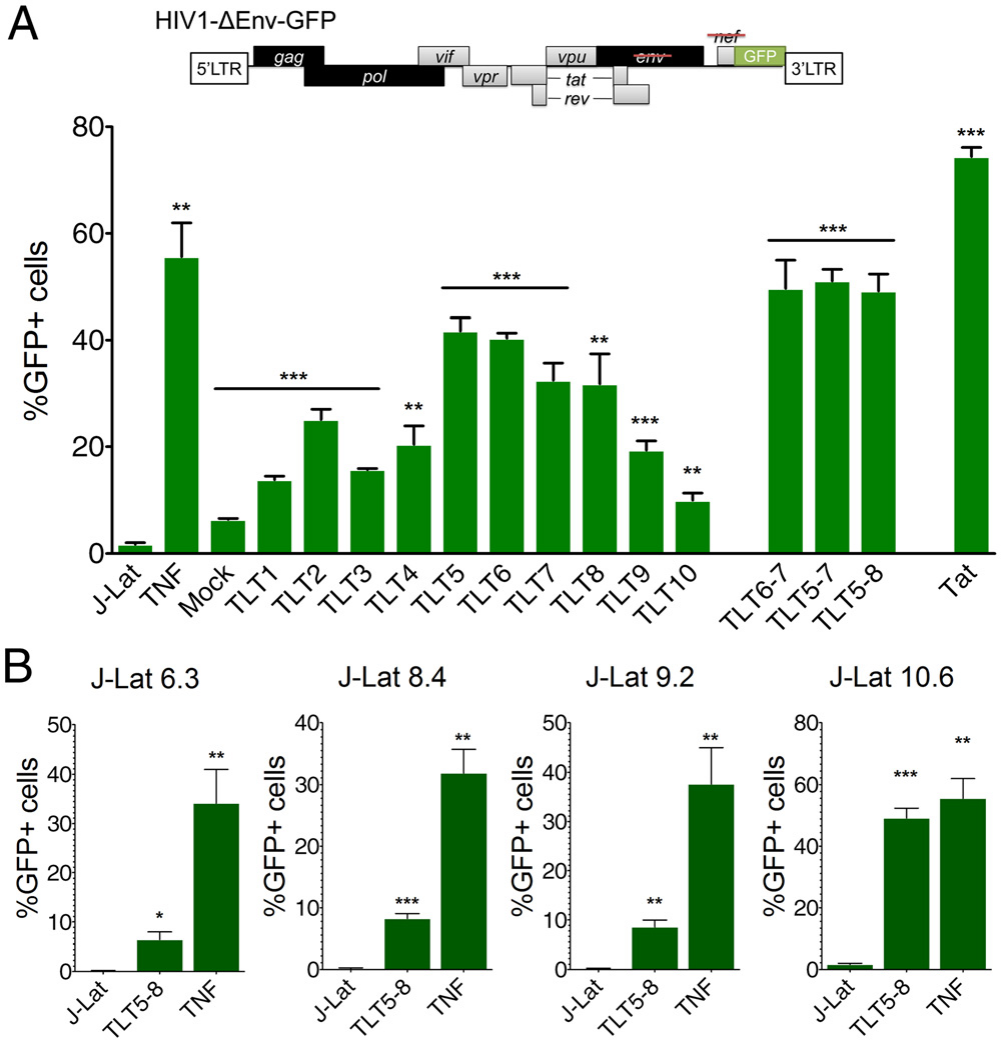
Reactivation of latent HIV-1 expression by TALE-TFs in multiple cell line models of HIV-1 latency. **(A) (Top)** Schematic representation of the HIV-1 proviral genome present in J-Lat cells. Full-length HIV-1 was derived from the molecular clone pNL4-3-ΔEnv-GFP and expresses a GFP gene from the LTR promoter. Structural viral genes are shown in black, auxiliary genes are shown in grey. The *nef* and *env* genes were inactivated to force a single infection cycle. **(Bottom)** Percentage of GFP positive J-Lat 10.6 cells after nucleofection with TALE-TF and Tat expression plasmids, or treatment with TNF-α (10 ng/μL). GFP positive cells were measured by flow cytometry 48 h after nucleofection. “J-Lat” indicates non-transfected J-Lat 10.6 cells. “Mock” indicates cells transfected with an empty pcDNA backbone. Error bars indicate standard deviation of three independent experiments (*n* = 3). **(B)** Percentage of GFP positive J-Lat 6.3, 8.4 and 9.2 cells after nucleofection with TLT5-8 and Tat expression plasmids, or treatment with TNF-α (10 ng/μL). GFP positive cells were measured by flow cytometry 48 h after nucleofection. “J-Lat” indicates non-nucleofected cells. Error bars indicate standard deviation (*n* = 3; *p < 0.05; **p < 0.01; ***p < 0.001; *t*-test sample vs control (J-Lat)).

We next asked whether TALE transcription factors could reactivate viral expression in a cell line model of HIV-1 latency. To explore this, we used the Jurkat-derived J-Lat lymphocytic cell lines, which harbor a full-length integrated HIV-1 proviral genome containing a GFP gene that serves as a reporter for viral gene expression (HIV1-ΔEnv-GFP) (Fig 3A). J-Lat cells poorly express the integrated proviruses under normal conditions, but viral gene expression can be efficiently induced by stimulation using tumor necrosis factor (TNF)-α [16]. Since each J-Lat clone is derived from a unique HIV-1 integration event, they display differential levels of gene and/or chromatin repression, as demonstrated by their distinct gene activation thresholds after TNF-α stimulation [85].

We nucleofected J-Lat 10.6 cells, which display a low viral gene activation threshold, with expression vectors encoding TALE transcription factors or Tat and evaluated HIV-1 expression by measuring the percentage of GFP-positive cells by flow cytometry (Fig 3A). As expected, cells treated with TNF-α or transfected with Tat showed robust reactivation, with upwards of 55% and 75% of cells producing GFP, respectively. Among all individual TALE transcription factors tested, TLT5 and 6 (~40% GFP-positive cells each) and TLT7 and 8 (~30% GFP-positive cells each) yielded the highest levels of expression, with cells transfected with TLT5-8 also showing upwards of 50% GFP-positive cells (*p* < 0.05) (Fig 3A). Nucleofection of an empty vector (pcDNA) resulted in minor (~5%) reactivation, indicating that stress from the nucleofection process can also contribute to reactivation (Fig 3A).

The relative potencies of the TALE activators in J-Lat 10.6 cells correlated with their ability to stimulate transcription in the reporter assay used in Fig 2. Analysis of mean fluorescence intensity (MFI) in transfected J-Lat 10.6 cells further indicated that each TALE activator induced approximately a 10 to 15-fold increase in viral gene expression (S1A Fig). Interestingly, in contrast to the episomal studies presented in Fig 2, TLT5-8 yielded a similar number of GFP positive cells as the combinations TLT6-7 and TLT5-7 (Fig 3A). One possible explanation for this is that measuring the number of GFP positive J-Lat 10.6 cells may not necessarily afford the amount of sensitivity needed to distinguish between the potencies of specific combinations of activators. Indeed, analysis of MFI in J-Lat 10.6 cells revealed that TLT5-8 induced higher amounts of viral gene expression (~20-fold compared to the negative control) than TLT7 or the combinations TLT6-7 and TLT5-7 (~14-fold compared to the negative control) (S1B Fig).

To test the versatility of the TALE transcription factors, we next evaluated their ability to induce HIV-1 transcription in the J-Lat clones 6.3, 8.4 and 9.2, which each possess a higher gene activation threshold than J-Lat 10.6 cells [16]. Sequence mapping of these clones previously revealed that the HIV-1 provirus is integrated into actively transcribed genes, unfavorable to HIV transcription [18,86]. We observed significant reactivation in each cell line tested after co-transfection with TLT5-8 (*p* < 0.05) but at rates much lower than by TNF-α stimulation (Fig 3B), indicating that the level of repression within latently-infected cells can influence the ability of TALEs to mediate activation.

### Combining TALE transcription factors with an HDAC inhibitor enhances latent HIV activation

We next explored the possibility of enhancing HIV-1 reactivation by combining TALE transcription factors with a histone deacetylase (HDAC) inhibitor. Because HIV-1 proviral integration in J-Lat clones favors heterochromatic regions, especially those near alphoid DNA repeat elements [16], we hypothesized that chromatin remodeling by HDAC inhibition could enhance TALE binding to the HIV-1 LTR, thereby increasing viral gene expression. Indeed, previous reports have indicated that the LTR promoter is typically hypoacetylated and that treatment with HDAC inhibitors can lead to the recruitment of the transcriptional machinery to the HIV-1 promoter [23], as well as activation of the positive transcription elongation factor b (P-TEFb), which can stimulate viral transcriptional elongation [87]. Moreover, multiple studies have shown that combining HDAC inhibitors with other compounds also capable of reversing HIV latency can synergistically increase viral reactivation across a variety of repression states [88–91].

**Fig 4:**
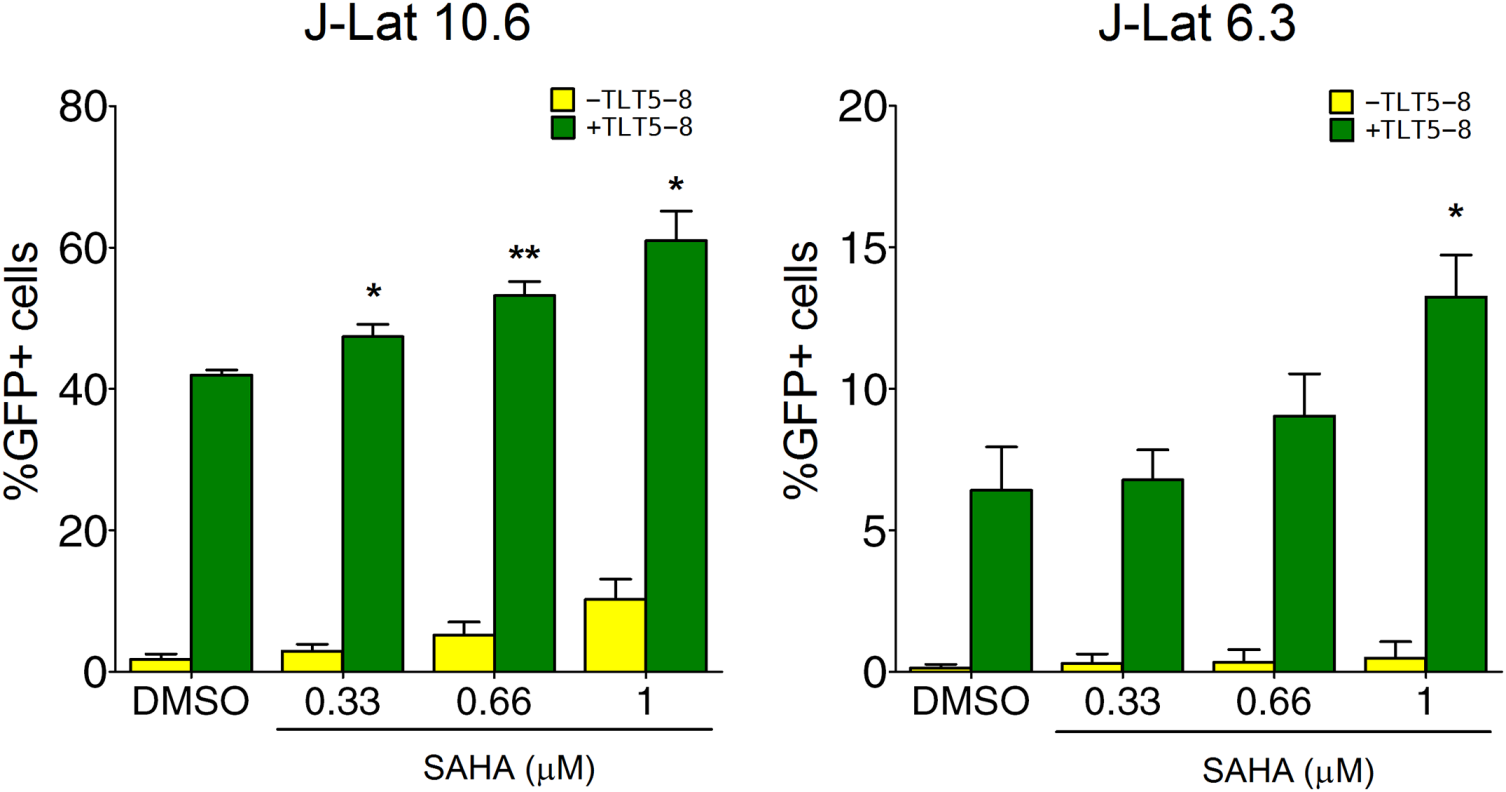
Enhanced reactivation of latent HIV-1 expression by combining TALE-TFs with a histone deacetylase inhibitor. Percentage of GFP-positive J-Lat 10.6 and 6.3 cells after nucleofection with TLT5-8 expression plasmids and treatment with increasing concentrations of SAHA or DMSO (0.1%) for 24 h. GFP-positive cells were measured by flow cytometry 48 h after nucleofection. Error bars indicate standard error of the mean of three independent experiments (*n* = 3; *p < 0.05; **p < 0.01; *t*-test).

We transfected J-Lat 10.6 and 6.3 cells, which each display distinct activation thresholds, with TLT5-8 and treated each population with 0.33, 0.66 or 1.0 μM of Vorinostat (i.e., suberoylanilide hydroxamic acid or SAHA), an HDAC inhibitor used for the treatment of malignant cancers but also capable of inducing expression of latent HIV-1 [29–31,92,93]. An initial screen led us to identify this specific range of SAHA concentrations (i.e. those that stimulate minimal amounts of HIV-1 transcription and induce low cell death) (S2 Fig). SAHA, in particular, is an FDA-approved inhibitor of Class I HDAC isotypes that has been shown to induce viral transcription in latent CD4^+^ T cells from HIV-infected patients [30,31] (though it was unable to increase HIV-1 production [30]). Compared to cells transfected with TLT5-8 only, we observed a significant increase (*p* < 0.05) in HIV-1 expression upon co-treatment with 1 μM SAHA (Fig 4). Specifically, reactivation was evident in up 65% and 15% of J-Lat 10.6 and 6.3 cells, respectively, corresponding to a 1.5- and 2-fold increase in HIV-1 transcription (Fig 4). We also observed increased levels of HIV-1 transcription in J-Lat 8.4 and 9.2 cells co-treated with SAHA and TLT5-8, but these values were neither neither significant nor dose-dependent (data now shown). Analysis of MFI in treated J-Lat 10.6 cells also revealed a significant and dose-dependent increase in viral gene expression after co-treatment with TLT5-8 and SAHA (S3 Fig). Collectively, these results demonstrate that complementing TALE transcription factors with HDAC inhibitors can lead to enhanced reactivation of latent HIV-1 expression.

## Discussion

HIV-1 latency is a substantial obstacle facing its eradication. Many approaches have been developed to indirectly activate HIV-1 from persistent cellular reservoirs, typically by altering the transcriptional landscape surrounding the integrated provirus [24–31]. While promising, these strategies have been unable to completely purge all virus from the reservoir and, in some cases, have even been associated with adverse effects, including immune reactions [32–35]. While targeted nucleases and recombinases have the capacity to excise integrated proviral DNA from infected cells [60–62,94], these tools also have the potential to induce unwanted non-specific DNA breaks, and thus carry a substantial risk of genotoxicity [39]. Conversely, synthetic transcription factors, which can be designed to induce transcription from the native viral promoter, represent a potentially safe and effective genetic alternative for reactivating latent virus in cells.

Here, we designed ten TALE proteins that spanned nearly the entire length of the HIV-1 LTR promoter in order to create activators capable of stimulating HIV-1 transcription. We identified four proteins (TLT5, TLT6, TLT7 and TLT8) that induced viral gene expression in cell line models of HIV latency. Interestingly, we observed that the effectiveness of individual TALEs correlated with their proximity to the TSS, as they targeted a conserved segment of the HIV LTR modulatory region, located upstream of the NF-κB and Sp1 *cis*-regulatory sites, and nearby regulatory elements that contribute to viral transcriptional initiation [82,95]. This data indicate that cooperation between endogenous transcription factors and engineered TALE activators may be an important factor for efficient reactivation of viral gene expression. These TALEs might thus promote transcription in a manner that mimics the natural activity of enhancer-like regulatory proteins, potentially serving as “molecular switches” for reactivation.

We showed that co-transfection of combinations of TALE transcription factors can further increase gene expression, indicating that strategies for mimicking the natural complexity of gene regulation [77,78] are also effective for inducing viral gene expression. Specifically, co-transfection of J-Lat 10.6 cells with TLT5-8 led to similar amounts of HIV-1 expression as those previously reported for compounds such as phytohemagglutinin (PHA), phorbolmyristate acetate (PMA) and prostratin [96]. Moreover, the specificity of our TALE activators could be improved in the future by incorporating recently described chemical- [68,97] or light-inducible [47,79] features that enable spatial and temporal control of HIV-1 expression.

Two reports initially demonstrated that engineered zinc-finger and TALE transcription factors can induce latent HIV expression, albeit with relatively modest efficiencies [98,99]. More recently, several other studies have shown that TALE [100] and CRISPR-Cas9 [101–103] activators can induce robust activation of latent viral expression. Our data correlates with these most recent reports and further indicates the potential of artificial transcription factor technology for eliminating the latent reservoir.

Although the TALE activators used in our study induced efficient HIV-1 expression in J-Lat 10.6 cells, reduced levels of viral gene expression were observed in J-Lat clones containing more repressive transcriptional backgrounds. Specifically, integration of the HIV-1 provirus into condensed regions of heterochromatin can negatively affect viral gene expression by hindering DNA accessibility to key host transcription factors [8,9]. Because histone deacetylases (HDACs) play a central role in maintaining HIV latency by promoting compact chromatin structures around integrated proviral DNA [8], we hypothesized that treating cells with HDAC inhibitors could increase binding site accessibility and further enhance HIV-1 reactivation. We found that combining TALE transcription factors with SAHA led to a significant increase in viral gene expression in J-Lat 6.3 and 10.6 cells comparable to those previously described for other latency-reversing compounds, including PHA and Bryostatin [96]. This indicates the broad utility of this concept for HIV-1 reactivation, and supports further investigation into the effects of combining HDAC inhibition with TALE-mediated activation in primary cell models of HIV latency [96]. Combining synthetic transcription factors and HDAC inhibition with HAART or nuclease-induced knockout of HIV co-receptors CCR5 and CXCR4 may also prove effective for combating HIV infection.

While TALE transcription factors have the capacity to activate latent HIV-1 transcription, several barriers must be overcome in order for this technology to be implemented for therapeutic purposes. In particular, due to their highly repetitive nature, lentiviral vector-mediated delivery of TALEs into cells has proven challenging [39,104,105]. Methods for overcoming this limitation are rapidly emerging, including those based on adenoviral [106,107], mRNA [108] and protein-based delivery systems [109,110]. Additionally, recent work has indicated that TALE nucleases could be introduced into cells as mRNA using lentivirus particles containing inactivated reverse transcriptase [111]. However, it remains unknown whether such systems can support *in vivo* delivery to latent resting CD4^+^ T cells. Although our current study indicates that a specific combination of four TALE activators is optimal for inducing HIV-1 transcriptional activation, current evidence demonstrate that the potency of these activators can be further enhanced to promote single TALE systems [112].

In summary, we demonstrate that TALE transcription factors are effective tools for activating latent HIV expression and their use, alone or in combination with HDAC inhibitors, could pave the way for improved HIV therapies.

## Supporting Information

S1 Fig Analysis of GFP mean fluorescence intensity in J-Lat 10.6 cells nucleofected with TALE-TFs. Mean fluorescence intensity (MFI) of GFP expression in J-Lat 10.6 cells after nucleofection with a **(A)** single TALE activator (TLT5, TLT6, TLT7, and TLT8) or **(B)** a combination of TALE activators (TLT7, TLT6-7, TLT5-7, and TLT5-8). MFI was measured by flow cytometry 48 h after nucleofection. “J-Lat” indicates non-transfected J-Lat 10.6 cells. Histograms are representative of a single experiment from three independent replicates.
(TIF)

S2 Fig Cell viability of J-Lat 10.6 cells treated with SAHA. Cell viability and viral gene expression profiles of J-Lat 10.6 cells treated with increasing concentrations of SAHA or DMSO after 24 only. Cell viability was measured as percentage of viable cells as determined by forward and side scatter (FSS/SCC) gating during flow cytometry. Viral gene expression was determined by measuring the percentage of GFP positive cells by flow cytometry.
(TIF)

S3 Fig SAHA increases mean fluorescence intensity in J-Lat cells nucleofected with combinations of TALE transcription factors. Mean fluorescence intensity (MFI) of GFP expressionin J-Lat 10.6 cells nucleofected with TALE-TF and co-treated with SAHA.J-Lat 10.6 cells were nucleofected with TLT5-8 expression plasmids and treated with increasing concentrations of SAHA or DMSO only for 24 h. MFI was measured by flow cytometry 48 h after nucleofection. Histograms are representative of a single experiment from three independent replicates.
(TIF)

S1 Table TALE proteins sequences used in this study. TALE N-terminal domain is colored orange. TALE DNA-binding domain is colored blue. RVD residues are shown in red. Nuclear localization signal (NLS) sequenceis highlighted grey. VP64 domain is colored green. HA tag is colored purple.
(DOCX)

S2 Table Primer sequences for the construction of the luciferase reporter plasmids used in this study. TALE binding sites are underlined. Restriction sites are in bold.
(DOCX)

S3 Table Sequence conservation of the TALE transcription factor binding sites across HIV-1 subtype B strains. Data based on 2014 edition of the HIV Sequence Database (http://hiv-web.lanl.gov). Dashes indicate sequence identity between subtype strains. Dots indicate gaps in the HIV genome sequence.
(DOCX)

## References

[1] Hammer SM, Katzenstein DA, Hughes MD, Gundacker H, Schooley RT, Haubrich RH, et al A Trial Comparing Nucleoside Monotherapy with Combination Therapy in HIV-Infected Adults with CD4 Cell Counts from 200 to 500 per Cubic Millimeter. New England Journal of Medicine. 1996 pp. 1081–1090. 8813038 10.1056/NEJM199610103351501

[2] Perelson AS, Essunger P, Cao Y, Vesanen M, Hurley A, Saksela K, et al Decay characteristics of HIV-1-infected compartments during combination therapy. Nature. 1997;387: 188–191. 9144290 10.1038/387188a0

[3] Gulick RM, Mellors JW, Havlir D, Eron JJ, Gonzalez C, McMahon D, et al Treatment with indinavir, zidovudine, and lamivudine in adults with human immunodeficiency virus infection and prior antiretroviral therapy. The New England journal of medicine. 1997. 10.1056/NEJM199709113371102 9287228

[4] Chun TW, Davey RT, Engel D, Lane HC, Fauci AS. Re-emergence of HIV after stopping therapy. Nature. 1999;401: 874–875. 10553903 10.1038/44755

[5] Chun TW, Stuyver L, Mizell SB, Ehler LA, Mican JA, Baseler M, et al Presence of an inducible HIV-1 latent reservoir during highly active antiretroviral therapy. Proc Natl Acad Sci U S A. 1997;94: 13193–13197. 9371822 10.1073/pnas.94.24.13193 PMC24285

[6] Finzi D. Identification of a Reservoir for HIV-1 in Patients on Highly Active Antiretroviral Therapy. Science. 1997 pp. 1295–1300. 10.1126/science.278.5341.1295 9360927

[7] Davey RT Jr., Bhat N, Yoder C, Chun TW, Metcalf JA, Dewar R, et al HIV-1 and T cell dynamics after interruption of highly active antiretroviral therapy (HAART) in patients with a history of sustained viral suppression. ProcNatlAcadSciUSA. 1999;96: 15109–15114. 10.1073/pnas.96.26.15109 PMC24781 10611346

[8] Siliciano RF, Greene WC. HIV latency. Cold Spring Harb Perspect Med. 2011;1: a007096 10.1101/cshperspect.a007096 22229121 PMC3234450

[9] Lassen K, Han Y, Zhou Y, Siliciano J, Siliciano RF. The multifactorial nature of HIV-1 latency. Trends Mol Med. 2004;10: 525–31. 15519278 10.1016/j.molmed.2004.09.006

[10] Ross EK, Buckler-White AJ, Rabson AB, Englund G, Martin MA. Contribution of NF-kappa B and Sp1 binding motifs to the replicative capacity of human immunodeficiency virus type 1: distinct patterns of viral growth are determined by T-cell types. J Virol. 1991;65: 4350–8. 2072454 10.1128/jvi.65.8.4350-4358.1991 PMC248874

[11] Sune C, Garcia-Blanco MA. Sp1 transcription factor is required for in vitro basal and Tat-activated transcription from the human immunodeficiency virus type 1 long terminal repeat. J Virol. 1995;69: 6572–6576. 7666561 10.1128/jvi.69.10.6572-6576.1995 PMC189562

[12] Yang Z, Zhu Q, Luo K ZQ. The 7SK small nuclear RNA inhibits the CDK9/cyclin T1 kinase to control transcription. Nature. 2001;414: 317–322. 11713532 10.1038/35104575

[13] Liou L-Y, Herrmann CH, Rice AP. Transient induction of cyclin T1 during human macrophage differentiation regulates human immunodeficiency virus type 1 Tat transactivation function. J Virol. 2002;76: 10579–10587. 12368300 10.1128/JVI.76.21.10579-10587.2002 PMC136632

[14] Karn J. The molecular biology of HIV latency: breaking and restoring the Tat-dependent transcriptional circuit. Curr Opin HIV AIDS. 2011;6: 4–11. 10.1097/COH.0b013e328340ffbb 21242887 PMC3032057

[15] Jordan A, Defechereux P, Verdin E. The site of HIV-1 integration in the human genome determines basal transcriptional activity and response to Tat transactivation. EMBO J. 2001;20: 1726–1738. 11285236 10.1093/emboj/20.7.1726 PMC145503

[16] Jordan A, Bisgrove D, Verdin E. HIV reproducibly establishes a latent infection after acute infection of T cells in vitro. 2003;22: 1868–1877. 10.1093/emboj/cdg188 PMC154479 12682019

[17] Greger IH, Demarchi F, Giacca M, Proudfoot NJ. Transcriptional interference perturbs the binding of Sp1 to the HIV-1 promoter. Nucleic Acids Res. 1998;26: 1294–1300. 9469840 10.1093/nar/26.5.1294 PMC147389

[18] Lenasi T, Contreras X, Peterlin BM. Transcriptional Interference Antagonizes Proviral Gene Expression to Promote HIV Latency. Cell Host Microbe. 2008;4: 123–133. 10.1016/j.chom.2008.05.016 18692772 PMC4217705

[19] Han Y, Lin YB, An W, Xu J, Yang HC, O’Connell K, et al Orientation-Dependent Regulation of Integrated HIV-1 Expression by Host Gene Transcriptional Readthrough. Cell Host Microbe. 2008;4: 134–146. 10.1016/j.chom.2008.06.008 18692773 PMC2604135

[20] Shan L, Yang H-C, Rabi SA, Bravo HC, Shroff NS, Irizarry RA, et al Influence of host gene transcription level and orientation on HIV-1 latency in a primary-cell model. J Virol. 2011;85: 5384–5393. 10.1128/JVI.02536-10 21430059 PMC3094997

[21] Kauder SE, Bosque A, Lindqvist A, Planelles V, Verdin E. Epigenetic regulation of HIV-1 latency by cytosine methylation. PLoS Pathog. 2009;5: e1000495 10.1371/journal.ppat.1000495 19557157 PMC2695767

[22] Blazkova J, Trejbalova K, Gondois-Rey F, Halfon P, Philibert P, Guiguen A, et al CpG methylation controls reactivation of HIV from latency. PLoS Pathog. 2009;5: e1000554 10.1371/journal.ppat.1000554 19696893 PMC2722084

[23] Williams SA, Chen L-F, Kwon H, Ruiz-Jarabo CM, Verdin E, Greene WC. NF-kappaB p50 promotes HIV latency through HDAC recruitment and repression of transcriptional initiation. EMBO J. 2006;25: 139–149. 16319923 10.1038/sj.emboj.7600900 PMC1356344

[24] Managlia EZ, Landay A, Al-Harthi L. Interleukin-7 induces HIV replication in primary naive T cells through a nuclear factor of activated T cell (NFAT)-dependent pathway. Virology. 2006;350: 443–452. 16542695 10.1016/j.virol.2006.02.019

[25] Prins JM, Jurriaans S, van Praag RM, Blaak H, van Rij R, Schellekens PT, et al Immuno-activation with anti-CD3 and recombinant human IL-2 in HIV-1-infected patients on potent antiretroviral therapy. AIDS. 1999;13: 2405–2410. 10597782 10.1097/00002030-199912030-00012

[26] Kulkosky J, Culnan DM, Roman J, Dornadula G, Schnell M, Boyd MR, et al Prostratin: Activation of latent HIV-1 expression suggests a potential inductive adjuvant therapy for HAART. Blood. 2001;98: 3006–3015. 11698284 10.1182/blood.v98.10.3006

[27] Korin YD, Brooks DG, Brown S, Korotzer A, Zack JA. Effects of prostratin on T-cell activation and human immunodeficiency virus latency. J Virol. 2002;76: 8118–8123. 12134017 10.1128/JVI.76.16.8118-8123.2002 PMC155166

[28] Lehrman G, Hogue IB, Palmer S, Jennings C, Spina CA, Wiegand A, et al Depletion of latent HIV-1 infection in vivo: A proof-of-concept study. Lancet. 2005;366: 549–555. 16099290 10.1016/S0140-6736(05)67098-5 PMC1894952

[29] Contreras X, Schweneker M, Chen C-S, McCune JM, Deeks SG, Martin J, et al Suberoylanilide hydroxamic acid reactivates HIV from latently infected cells. J Biol Chem. 2009;284: 6782–6789. 10.1074/jbc.M807898200 19136668 PMC2652322

[30] Archin NM, Liberty AL, Kashuba AD, Choudhary SK, Kuruc JD, Crooks AM. Administration of vorinostat disrupts HIV-1 latency in patients on antiretroviral therapy. Nature. 2013;487: 482–485. 10.1038/nature11286 PMC3704185 22837004

[31] Elliott JH, Wightman F, Solomon A, Ghneim K, Ahlers J, Cameron MJ, et al Activation of HIV transcription with short-course vorinostat in HIV-infected patients on suppressive antiretroviral therapy. PLoS Pathog. 2014;10: e1004473 10.1371/journal.ppat.1004473 25393648 PMC4231123

[32] Williams S a, Greene WC. Regulation of HIV-1 latency by T-cell activation. Cytokine. 2007;39: 63–74. 17643313 10.1016/j.cyto.2007.05.017 PMC2063506

[33] Richman DD, Margolis DM, Delaney M, Greene WC, Hazuda D, Pomerantz RJ. The challenge of finding a cure for HIV infection. Science. 2009;323: 1304–7. 10.1126/science.1165706 19265012

[34] Dahl V, Josefsson L, Palmer S. HIV reservoirs, latency, and reactivation: Prospects for eradication. Antiviral Research. 2010 pp. 286–294. 10.1016/j.antiviral.2009.09.016 19808057

[35] Chomont N, El-Far M, Ancuta P, Trautmann L, Procopio FA, Yassine-Diab B, et al HIV reservoir size and persistence are driven by T cell survival and homeostatic proliferation. Nat Med. 2009;15: 893–900. 10.1038/nm.1972 19543283 PMC2859814

[36] Gersbach CA, Gaj T, Barbas CF. Synthetic Zinc Finger Proteins: The Advent of Targeted Gene Regulation and Genome Modification Technologies. Acc Chem Res. 2014;47: 2309–18. 10.1021/ar500039w 24877793 PMC4139171

[37] Doyle EL, Stoddard BL, Voytas DF, Bogdanove AJ. TAL effectors: Highly adaptable phytobacterial virulence factors and readily engineered DNA-targeting proteins. Trends in Cell Biology. 2013 pp. 390–398. 10.1016/j.tcb.2013.04.003 23707478 PMC3729746

[38] Hsu PD, Lander ES, Zhang F. Development and applications of CRISPR-Cas9 for genome engineering. Cell. Cell Press; 2014;157: 1262–1278. 10.1016/j.cell.2014.05.010 PMC4343198 24906146

[39] Gaj T, Gersbach CA, Barbas CF III. ZFN, TALEN and CRISPR/Cas-based methods for genome engineering. Trends Biotechol. 2014;31: 397–405. 10.1016/j.tibtech.2013.04.004 PMC3694601 23664777

[40] Miller JC, Tan S, Qiao G, Barlow K a, Wang J, Xia DF, et al A TALE nuclease architecture for efficient genome editing. Nat Biotechnol. 2011;29: 143–8. 10.1038/nbt.1755 21179091

[41] Zhang F, Cong L, Lodato S, Kosuri S, Church G, Arlotta P. Programmable Sequence-Specific Transcriptional Regulation of Mammalian Genome Using Designer TAL Effectors. Nat Biotechnol. 2011;29: 149–153. 21248753 10.1038/nbt.1775 PMC3084533

[42] Cong L, Zhou R, Kuo Y, Cunniff M, Zhang F. Comprehensive interrogation of natural TALE DNA-binding modules and transcriptional repressor domains. Nature Communications. 2012 p. 968 10.1038/ncomms1962 22828628 PMC3556390

[43] Reyon D, Tsai SQ, Khayter C, Foden JA, Sander JD, Joung JK. FLASH assembly of TALENs for high-throughput genome editing. Nature Biotechnology. 2012 pp. 460–465. 10.1038/nbt.2170 22484455 PMC3558947

[44] Mussolino C, Morbitzer R, Lütge F, Dannemann N, Lahaye T, Cathomen T. A novel TALE nuclease scaffold enables high genome editing activity in combination with low toxicity. Nucleic Acids Res. 2011;39: 9283–93. 10.1093/nar/gkr597 21813459 PMC3241638

[45] Mercer AC, Gaj T, Fuller RP, Barbas CF. Chimeric TALE recombinases with programmable DNA sequence specificity. Nucleic Acids Res. 2012;40: 11163–11172. 10.1093/nar/gks875 23019222 PMC3510496

[46] Maeder ML, Angstman JF, Richardson ME, Linder SJ, Cascio VM, Tsai SQ, et al Targeted DNA demethylation and activation of endogenous genes using programmable TALE-TET1 fusion proteins. Nat Biotechnol. 2013;31: 1137–42. 10.1038/nbt.2726 24108092 PMC3858462

[47] Konermann S, Brigham MD, Trevino AE, Hsu PD, Heidenreich M, Cong L, et al Optical control of mammalian endogenous transcription and epigenetic states. Nature. Nature Publishing Group; 2013;500: 472–6. 10.1038/nature12466 PMC3856241 23877069

[48] Hilton IB, D’Ippolito AM, Vockley CM, Thakore PI, Crawford GE, Reddy TE, et al Epigenome editing by a CRISPR-Cas9-based acetyltransferase activates genes from promoters and enhancers. Nat Biotechnol. 2015;33: 510–7. 10.1038/nbt.3199 25849900 PMC4430400

[49] Boch J, Scholze H, Schornack S, Landgraf A, Hahn S, Kay S, et al Breaking the code of DNA binding specificity of TAL-type III effectors. Science. 2009;326: 1509–1512. 10.1126/science.1178811 19933107

[50] Moscou MJ, Bogdanove AJ. A simple cipher governs DNA recognition by TAL effectors. Science. 2009;326: 1501 10.1126/science.1178817 19933106

[51] Mussolino C, Cathomen T. TALE nucleases: tailored genome engineering made easy. Curr Opin Biotechnol. Elsevier Ltd; 2012;23: 644–50. 10.1016/j.copbio.2012.01.013 22342754

[52] Cermak T, Doyle EL, Christian M, Wang L, Zhang Y, Schmidt C, et al Efficient design and assembly of custom TALEN and other TAL effector-based constructs for DNA targeting. Nucleic Acids Res. 2011;39: e82 10.1093/nar/gkr218 21493687 PMC3130291

[53] Reynolds L, Ullman C, Moore M, Isalan M, West MJ, Clapham P, et al Repression of the HIV-1 5’ LTR promoter and inhibition of HIV-1 replication by using engineered zinc-finger transcription factors. Proc Natl Acad Sci U S A. 2003;100: 1615–1620. 12574502 10.1073/pnas.252770699 PMC149881

[54] Segal DJ, Gonçalves J, Eberhardy S, Swan CH, Torbett BE, Li X, et al Attenuation of HIV-1 replication in primary human cells with a designed zinc finger transcription factor. J Biol Chem. 2004;279: 14509–19. 14734553 10.1074/jbc.M400349200

[55] Kim Y-S, Kim J-M, Jung D-L, Kang J-E, Lee S, Kim JS, et al Artificial zinc finger fusions targeting Sp1-binding sites and the trans-activator-responsive element potently repress transcription and replication of HIV-1. J Biol Chem. 2005;280: 21545–52. 15743774 10.1074/jbc.M414136200

[56] Eberhardy SR, Goncalves J, Coelho S, Segal DJ, Berkhout B, Iii CFB. Inhibition of Human Immunodeficiency Virus Type 1 Replication with Artificial Transcription Factors Targeting the Highly Conserved Primer-Binding Site. J Virol. 2006;80: 2873–2883. 16501096 10.1128/JVI.80.6.2873-2883.2006 PMC1395442

[57] Coburn GA, Cullen BR. Potent and specific inhibition of human immunodeficiency virus type 1 replication by RNA interference. J Virol. 2002;76: 9225–9231. 12186906 10.1128/JVI.76.18.9225-9231.2002 PMC136455

[58] Kohn DB, Bauer G, Rice CR, Rothschild JC, Carbonaro DA, Valdez P, et al A clinical trial of retroviral-mediated transfer of a rev-responsive element decoy gene into CD34(+) cells from the bone marrow of human immunodeficiency virus-1-infected children. Blood. 1999;94: 368–371. 10381536

[59] Dave RS, Pomerantz RJ. Antiviral effects of human immunodeficiency virus type 1-specific small interfering RNAs against targets conserved in select neurotropic viral strains. J Virol. 2004;78: 13687–13696. 15564478 10.1128/JVI.78.24.13687-13696.2004 PMC533941

[60] Qu X, Wang P, Ding D, Li L, Wang H, Ma L, et al Zinc-finger-nucleases mediate specific and efficient excision of HIV-1 proviral DNA from infected and latently infected human T cells. Nucleic Acids Res. 2013;41: 7771–7782. 10.1093/nar/gkt571 23804764 PMC3763554

[61] Ebina H, Misawa N, Kanemura Y, Koyanagi Y. Harnessing the CRISPR/Cas9 system to disrupt latent HIV-1 provirus. Sci Rep. 2013;3: 2510 10.1038/srep02510 23974631 PMC3752613

[62] Ebina H, Kanemura Y, Misawa N, Sakuma T, Kobayashi T, Yamamoto T, et al A High Excision Potential of TALENs for Integrated DNA of HIV-Based Lentiviral Vector. PLoS One. 2015;10: e0120047 10.1371/journal.pone.0120047 25781496 PMC4363575

[63] Perez EE, Wang J, Miller JC, Jouvenot Y, Kim KA, Liu O, et al Establishment of HIV-1 resistance in CD4+ T cells by genome editing using zinc-finger nucleases. Nat Biotechnol. 2008;26: 808–816. 10.1038/nbt1410 18587387 PMC3422503

[64] Tebas P, Stein D, Tang WW, Frank I, Wang SQ, Lee G, et al Gene editing of CCR5 in autologous CD4 T cells of persons infected with HIV. N Engl J Med. 2014;370: 901–10. 10.1056/NEJMoa1300662 24597865 PMC4084652

[65] Wang W, Ye C, Liu J, Zhang D, Kimata JT, Zhou P. CCR5 Gene Disruption via Lentiviral Vectors Expressing Cas9 and Single Guided RNA Renders Cells Resistant to HIV-1 Infection. PLoS One. 2014;9: e115987 10.1371/journal.pone.0115987 25541967 PMC4277423

[66] Polstein LR, Perez-pinera P, Kocak DD, Vockley CM, Song L, Safi A, et al Genome-Wide Specificity of DNA-Binding, Gene Regulation, and Chromatin Remodeling by TALE- and CRISPR / Cas9-Based Transcriptional Activators. Genome Res. 2015;25: 1158–69. 10.1101/gr.179044.114 26025803 PMC4510000

[67] Adachi A, Gendelman HE, Koenig S, Folks T, Willey R, Rabson A, et al Production of acquired immunodeficiency syndrome-associated retrovirus in human and nonhuman cells transfected with an infectious molecular clone. J Virol. 1986;59: 284–291. 3016298 10.1128/jvi.59.2.284-291.1986 PMC253077

[68] Mercer AC, Gaj T, Sirk SJ, Lamb BM, Barbas CF. Regulation of Endogenous Human Gene Expression by Ligand- Inducible TALE Transcription Factors. ACS Synth Biol. 2014;3: 723–30. 10.1021/sb400114p 24251925 PMC4097969

[69] Lamb BM, Mercer AC, Barbas CF. Directed evolution of the TALE N-terminal domain for recognition of all 5’ bases. Nucleic Acids Res. 2013;41: 9779–85. 10.1093/nar/gkt754 23980031 PMC3834825

[70] Jordan M, Schallhorn A, Wurm FM. Transfecting mammalian cells: Optimization of critical parameters affecting calcium-phosphate precipitate formation. Nucleic Acids Res. 1996;24: 596–601. 8604299 10.1093/nar/24.4.596 PMC145683

[71] Gaj T, Sirk SJ, Tingle RD, Mercer AC, Wallen MC, Barbas CF. Enhancing the specificity of recombinase-mediated genome engineering through dimer interface redesign. J Am Chem Soc. American Chemical Society; 2014;136: 5047–5056. 10.1021/ja4130059 PMC3985937 24611715

[72] Marks PA, Breslow R. Dimethyl sulfoxide to vorinostat: development of this histone deacetylase inhibitor as an anticancer drug. Nat Biotechnol. 2007;25: 84–90. 17211407 10.1038/nbt1272

[73] Beerli RR, Segal DJ, Dreier B, Barbas CF III. Toward controlling gene expression at will : Specific regulation of the erbB—2 HER—2 promoter by using polydactyl zinc finger. Proc Natl Acad Sci. 1998;95: 14628–14633. 9843940 10.1073/pnas.95.25.14628 PMC24500

[74] Konermann S, Brigham MD, Trevino a E, Joung J, Abudayyeh OO, Barcena C, et al Genome-scale transcriptional activation by an engineered CRISPR-Cas9 complex. Nature. Nature Publishing Group; 2014;517: 583–588. 10.1038/nature14136 PMC4420636 25494202

[75] Mali P, Aach J, Stranges PB, Esvelt KM, Moosburner M, Kosuri S, et al CAS9 transcriptional activators for target specificity screening and paired nickases for cooperative genome engineering. Nat Biotechnol. Nature Publishing Group; 2013;31: 833–8. 10.1038/nbt.2675 PMC3818127 23907171

[76] Perez-Pinera P, Kocak DD, Vockley CM, Adler AF, Kabadi AM, Polstein LR, et al RNA-guided gene activation by CRISPR-Cas9-based transcription factors. Nat Methods. 2013;10: 973–6. 10.1038/nmeth.2600 23892895 PMC3911785

[77] Perez-pinera P, Ousterout DG, Brunger JM, Farin AM, Glass KA, Guilak F, et al Synergistic and tunable human gene activation by combinations of synthetic transcription factors. Nat Methods. 2013;10: 239–242. 10.1038/nmeth.2361 23377379 PMC3719416

[78] Maeder ML, Linder SJ, Reyon D, Angstman JF, Fu Y, Sander JD, et al Robust, synergistic regulation of human gene expression using TALE activators. Nat Methods. 2013;10: 243–245. 10.1038/nmeth.2366 23396285 PMC3584229

[79] Polstein LR, Gersbach C a. A light-inducible CRISPR-Cas9 system for control of endogenous gene activation. Nat Chem Biol. 2015;11: 198–200. 10.1038/nchembio.1753 25664691 PMC4412021

[80] Choy B, Green MR. Eukaryotic activators function during multiple steps of preinitiation complex assembly. Nature. 1993;366: 531–536. 8255291 10.1038/366531a0

[81] Memedula S, Belmont AS. Sequential recruitment of HAT and SWI/SNF components to condensed chromatin by VP16. Curr Biol. 2003;13: 241–246. 12573221 10.1016/s0960-9822(03)00048-4

[82] Rohr O, Marban C, Aunis D, Schaeffer E. Regulation of HIV-1 gene transcription: from lymphocytes to microglial cells. J Leukoc Biol. 2003;74: 736–749. 12960235 10.1189/jlb.0403180

[83] Lau C-H, Zhu H, Tay JC-K, Li Z, Tay FC, Chen C, et al Genetic rearrangements of variable di-residue (RVD)-containing repeat arrays in a baculoviral TALEN system. Mol Ther—Methods Clin Dev. 2014;1: 14050 10.1038/mtm.2014.50 26015987 PMC4362386

[84] Karn J. Tat, a novel regulator of HIV transcription and latency. HIV Seq Compend. 2000; 2–18.

[85] Jordan A, Bisgrove D, Verdin E. HIV reproducibly establishes a latent infection after acute infection of T cells in vitro. 2003;22. 10.1093/emboj/cdg188 PMC154479 12682019

[86] Gallastegui E, Millán-Zambrano G, Terme J-M, Chávez S, Jordan A. Chromatin reassembly factors are involved in transcriptional interference promoting HIV latency. J Virol. 2011;85: 3187–3202. 10.1128/JVI.01920-10 21270164 PMC3067836

[87] Jamaluddin S, Hu P, Danels YJ, Siwak EB, Andrew P, Rice AP. The Broad Spectrum Histone Deacetylase Inhibitors Vorinostat and Panobinostat Activate Latent HIV in CD4 + T cells in part through Phosphorylation of the T-Loop of the CDK9 Subunit of P-TEFb.: 1–15. 10.1089/aid.2015.0347 PMC4761808 26727990

[88] Reuse S, Calao M, Kabeya K, Guiguen A, Gatot J-S, Quivy V, et al Synergistic activation of HIV-1 expression by deacetylase inhibitors and prostratin: implications for treatment of latent infection. PLoS One. 2009;4: e6093 10.1371/journal.pone.0006093 19564922 PMC2699633

[89] Pérez M, de Vinuesa AG, Sanchez-Duffhues G, Marquez N, Bellido ML, Muñoz-Fernandez MA, et al Bryostatin-1 synergizes with histone deacetylase inhibitors to reactivate HIV-1 from latency. Curr HIV Res. 2010;8: 418–29. ABS-86 [pii] 20636281 10.2174/157016210793499312

[90] Burnett JC, Lim K-I, Calafi A, Rossi JJ, Schaffer D V, Arkin AP. Combinatorial latency reactivation for HIV-1 subtypes and variants. J Virol. 2010;84: 5958–74. 10.1128/JVI.00161-10 20357084 PMC2876650

[91] Martínez-Bonet M, Isabel Clemente M, Jesús Serramía M, Muñoz E, Moreno S, Ángeles Muñoz-Fernández M. Synergistic Activation of Latent HIV-1 Expression by Novel Histone Deacetylase Inhibitors and Bryostatin-1. Sci Rep. Nature Publishing Group; 2015;5: 16445. 10.1038/srep16445 PMC4643323 26563568

[92] Rasmussen TA, Schmeltz Søgaard O, Brinkmann C, Wightman F, Lewin SR, Melchjorsen J, et al Comparison of HDAC inhibitors in clinical development: effect on HIV production in latently infected cells and T-cell activation. Hum Vaccin Immunother. 2013;9: 993–1001. 10.4161/hv.23800 23370291 PMC3899169

[93] Margolis DM. Histone deacetylase inhibitors and HIV latency. Curr Opin HIV AIDS. 2011;6: 25–29. 10.1097/COH.0b013e328341242d 21242890 PMC3079555

[94] Sarkar I, Hauber I, Hauber J, Buchholz F. HIV-1 proviral DNA excision using an evolved recombinase. Science. 2007;316: 1912–1915. 17600219 10.1126/science.1141453

[95] Pereira LA, Bentley K, Peeters A, Churchill MJ, Deacon NJ. A compilation of cellular transcription factor interactions with the HIV-1 LTR promoter. Nucleic Acids Res. 2000;28: 663–668. 10637316 10.1093/nar/28.3.663 PMC102541

[96] Spina C a, Anderson J, Archin NM, Bosque A, Chan J, Famiglietti M, et al An in-depth comparison of latent HIV-1 reactivation in multiple cell model systems and resting CD4+ T cells from aviremic patients. PLoS Pathog. 2013;9: e1003834 10.1371/journal.ppat.1003834 24385908 PMC3873446

[97] Zetsche B, Volz SE, Zhang F. A split-Cas9 architecture for inducible genome editing and transcription modulation. Nat Biotechnol. 2015; 9–11. 10.1038/nbt.3149 PMC4503468 25643054

[98] Wang P, Qu X, Wang X, Zhu X, Zeng H, Chen H, et al Specific reactivation of latent HIV-1 with designer zinc-finger transcription factors targeting the HIV-1 5’-LTR promoter. Gene Ther. 2014; 1–6. 10.1038/gt.2014.21 24622733

[99] Wang X, Wang P, Fu Z, Ji H, Qu X, Zeng H, et al Designed TALE proteins efficiently induced the expression of latent HIV-1 in latently infected cells. AIDS Res Hum Retroviruses. 2015;31: 98–106. 10.1089/AID.2014.0121 25403229 PMC4287188

[100] Geissler R, Hauber I, Funk N, Richter A, Behrens M, Renner I, et al Patient-adapted, specific activation of HIV-1 by customized TAL effectors (TALEs), a proof of principle study. Virology. Elsevier; 2015;486: 248–254. 10.1016/j.virol.2015.09.018 26474371

[101] Zhang Y, Yin C, Zhang T, Li F, Yang W, Kaminski R, et al CRISPR/gRNA-directed synergistic activation mediator (SAM) induces specific, persistent and robust reactivation of the HIV-1 latent reservoirs. Sci Rep. Nature Publishing Group; 2015;5: 16277 10.1038/srep16277 PMC4633726 26538064

[102] Saayman SM, Lazar DC, Scott TA, Hart JR, Takahashi M, Burnett JC, et al Potent and targeted activation of latent HIV-1 using the CRISPR/dCas9 activator complex. Mol Ther. 2015; 10.1038/mt.2015.202 PMC4786915 26581162

[103] Limsirichai P, Gaj T, Schaffer D V. CRISPR-mediated activation of latent HIV-1 expression. Mol Ther. 2015; 10.1038/mt.2015.213 PMC4786916 26607397

[104] Ain QU, Chung JY, Kim Y-H. Current and future delivery systems for engineered nucleases: ZFN, TALEN and RGEN. J Control Release. Elsevier B.V.; 2014; 10.1016/j.jconrel.2014.12.036 25553825

[105] Holkers M, Maggio I, Liu J, Janssen JM, Miselli F, Mussolino C, et al Differential integrity of TALE nuclease genes following adenoviral and lentiviral vector gene transfer into human cells. Nucleic Acids Res. 2013;41: e63 10.1093/nar/gks1446 23275534 PMC3597656

[106] Holkers M, Cathomen T, Gonçalves M a F V. Construction and characterization of adenoviral vectors for the delivery of TALENs into human cells. Methods. Elsevier Inc.; 2014;69: 179–187. 10.1016/j.ymeth.2014.02.017 24561826

[107] Holkers M, Maggio I, Henriques SFD, Janssen JM, Cathomen T, Gonçalves M a F V. Adenoviral vector DNA for accurate genome editing with engineered nucleases. Nat Methods. 2014; 10.1038/nmeth.3075 25152084

[108] Mock U, Machowicz R, Hauber I, Horn S, Abramowski P, Berdien B, et al mRNA transfection of a novel TAL effector nuclease (TALEN) facilitates efficient knockout of HIV co-receptor CCR5. Nucleic Acids Res. 2015;43: 5560–5571. 10.1093/nar/gkv469 25964300 PMC4477672

[109] Liu J, Gaj T, Patterson JT, Sirk SJ, Barbas CF. Cell-penetrating peptide-mediated delivery of TALEN proteins via bioconjugation for genome engineering. PLoS One. 2014;9: e85755 10.1371/journal.pone.0085755 24465685 PMC3896395

[110] Liu J, Gaj T, Yang Y, Wang N, Shui S, Kim S, et al Efficient delivery of nuclease proteins for genome editing in human stem cells and primary cells. Nat Protoc. 2015;10: 1842–59. 10.1038/nprot.2015.117 26492140

[111] Mock U, Riecken K, Berdien B, Qasim W, Chan E, Cathomen T, et al Novel lentiviral vectors with mutated reverse transcriptase for mRNA delivery of TALE nucleases. Sci Rep. 2014;4: 1–8. 10.1038/srep06409 PMC4166709 25230987

[112] Chavez A, Scheiman J, Vora S, Pruitt BW, Tuttle M, P R Iyer E, et al Highly efficient Cas9-mediated transcriptional programming. Nat Methods. Nature Publishing Group; 2015; 1–5. 10.1038/nmeth.3312 PMC4393883 25730490

