# Supplementary figures and images for: Reactivation of Latent HIV-1 Expression by Engineered TALE Transcription Factors
Source: PLoS One. 2016 Mar 2;11(3):e0150037. doi: 10.1371/journal.pone.0150037 (PMC4774903; doi:10.1371/journal.pone.0150037)

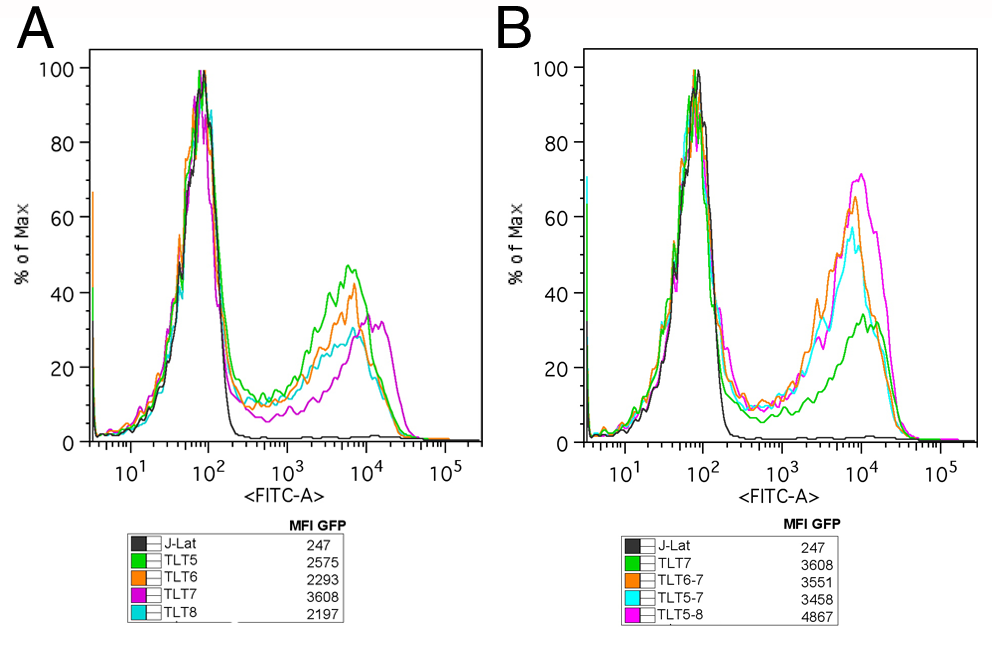

Supplement: S1 Fig — Mean fluorescence intensity (MFI) of GFP expression in J-Lat 10.6 cells after nucleofection with a (A) single TALE activator (TLT5, TLT6, TLT7, and TLT8) or (B) a combination of TALE activators (TLT7, TLT6-7, TLT5-7, and TLT5-8). MFI was measured by flow cytometry 48 h after nucleofection. “J-Lat” indicates non-transfected J-Lat 10.6 cells. Histograms are representative of a single experiment from three independent replicates. (TIF) [file pone.0150037.s001.tif]

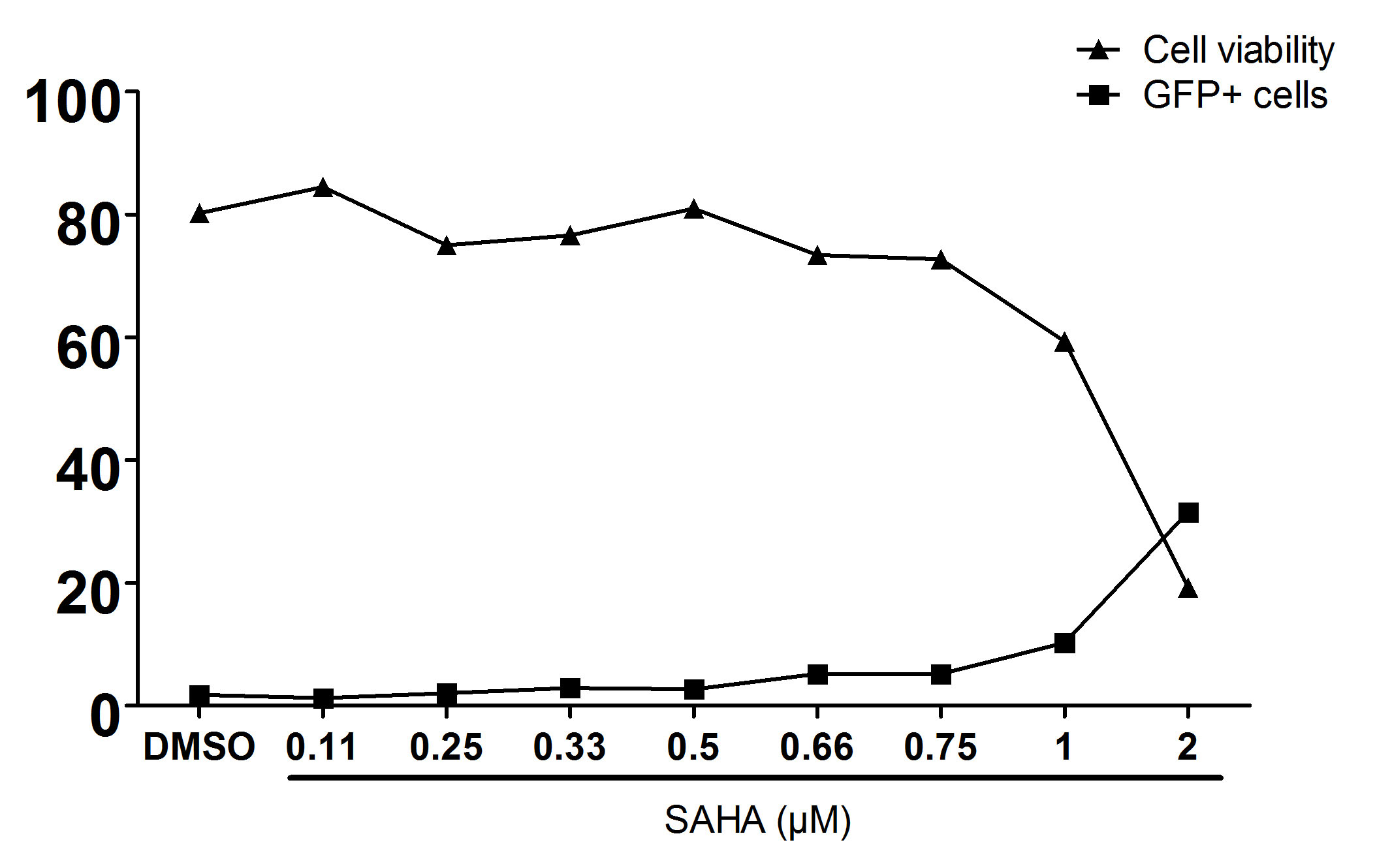

Supplement: S2 Fig — Cell viability and viral gene expression profiles of J-Lat 10.6 cells treated with increasing concentrations of SAHA or DMSO after 24 only. Cell viability was measured as percentage of viable cells as determined by forward and side scatter (FSS/SCC) gating during flow cytometry. Viral gene expression was determined by measuring the percentage of GFP positive cells by flow cytometry. (TIF) [file pone.0150037.s002.tif]

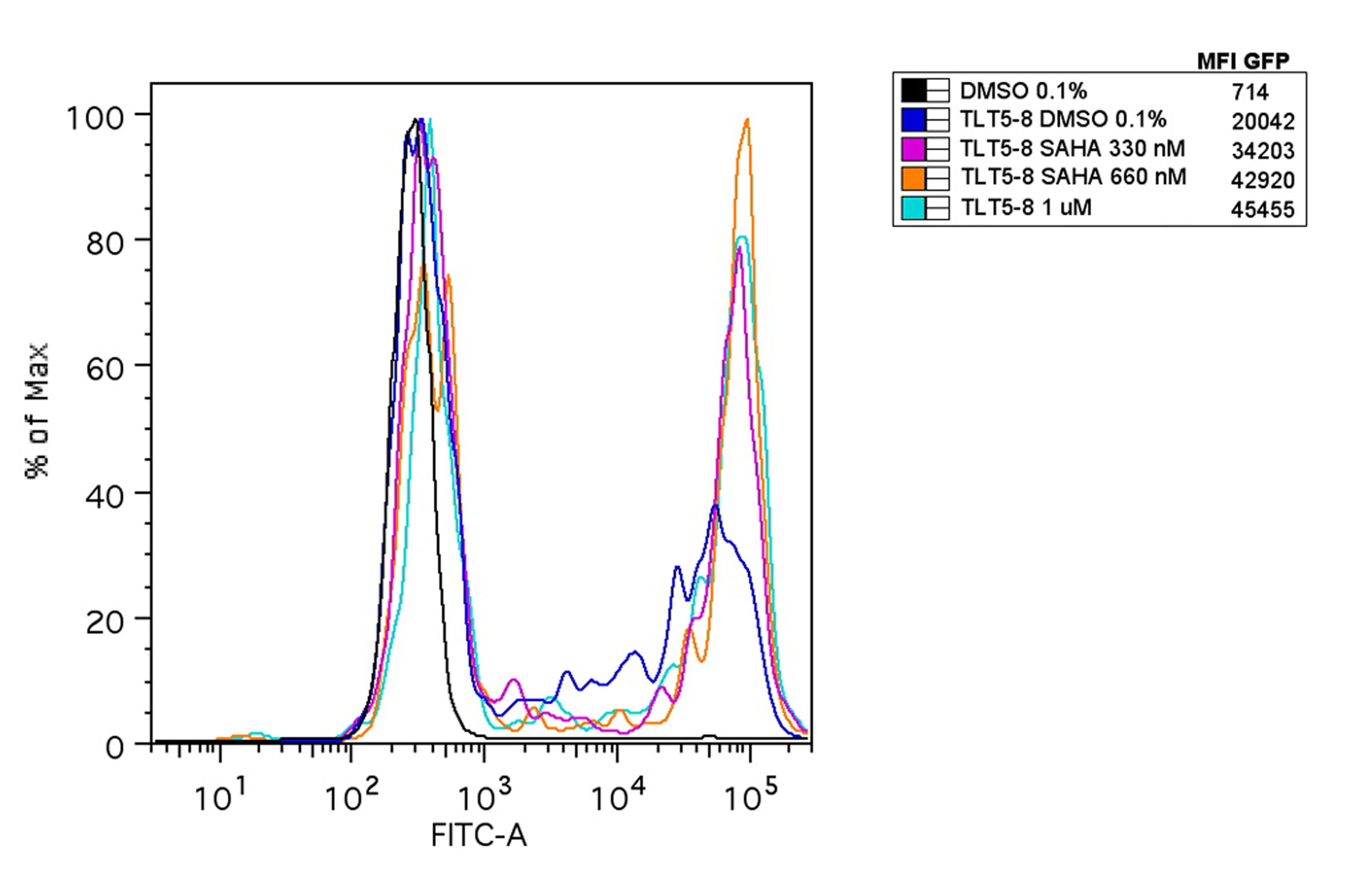

Supplement: S3 Fig — Mean fluorescence intensity (MFI) of GFP expressionin J-Lat 10.6 cells nucleofected with TALE-TF and co-treated with SAHA.J-Lat 10.6 cells were nucleofected with TLT5-8 expression plasmids and treated with increasing concentrations of SAHA or DMSO only for 24 h. MFI was measured by flow cytometry 48 h after nucleofection. Histograms are representative of a single experiment from three independent replicates. (TIF) [file pone.0150037.s003.tif]
