# Supplementary material for: Reactivation of Latent HIV-1 Expression by Engineered TALE Transcription Factors
Source: PLoS One. 2016 Mar 2;11(3):e0150037. doi: 10.1371/journal.pone.0150037 (PMC4774903; doi:10.1371/journal.pone.0150037)
Supplement: S1 Table — TALE N-terminal domain is colored orange. TALE DNA-binding domain is colored blue. RVD residues are shown in red. Nuclear localization signal (NLS) sequenceis highlighted grey. VP64 domain is colored green. HA tag is colored purple. (DOCX) [file pone.0150037.s004.docx]

**>TLT1-TF**

MAQAASGSPRPPRAKPAPRRRAAQPSDASPAAQVDLRTLGYSQQQQEKIKPKVRSTVAQHHEALVGHGFTHAHIVALSQHPAALGTVAVTYQHIITALPEATHEDIVGVGKQWSGARALEALLTDAGELRGPPLQLDTGQLVKIAKRGGVTAMEAVHASRNALTGAP

LNLTPDQVVAIASNIGGKQALETVQRLLPVLCQDH

GLTPDQVVAIASHDGGKQALETVQRLLPVLCQDH

GLTPDQVVAIASHDGGKQALETVQRLLAVLCQDH

GLTPDQVVAIASNIGGKQALETVQRLLAVLCQDH

GLTPDQVVAIASHDGGKQALETVQRLLAVLCQDH

GLTPDQVVAIASNIGGKQALETVQRLLAVLCQDH

GLTPDQVVAIASHDGGKQALETVQRLLPVLCQDH

GLTPDQVVAIASNIGGKQALETVQRLLPVLCQDH

GLTPDQVVAIASHDGGKQALETVQRLLPVLCQDH

GLTPDQVVAIASNIGGKQALETVQRLLPVLCQDH

GLTPDQVVAIASNIGGKQALETVQRLLPVLCQDH

GLTPDQVVAIASNNGGKQALETVQRLLPVLCQDH

GLTPDQVVAIASNNGGKQALETVQRLLPVLCQDH

GLTPDQVVAIASHDGGKQALETVQRLLPVLCQDH

GLTPDQVVAIASNGGGKQALESIVAQLSRPDPALAALTNDHLVALACLGGRPAMDAVKKGLPHAPELIRRVNRRIGERTSHRVADYAQVVRVLEFFQCHSHPAYAFDEAMTQFGMSGQAGQASPKKKRKVGRADALDDFDLDMLGSDALDDFDLDMLGSDALDDFDLDMLGSDALDDFDLDMLINYPYDVPDYAS

**>TLT2-TF**

MAQAASGSPRPPRAKPAPRRRAAQPSDASPAAQVDLRTLGYSQQQQEKIKPKVRSTVAQHHEALVGHGFTHAHIVALSQHPAALGTVAVTYQHIITALPEATHEDIVGVGKQWSGARALEALLTDAGELRGPPLQLDTGQLVKIAKRGGVTAMEAVHASRNALTGAP

LNLTPDQVVAIASNNGGKQALETVQRLLPVLCQDH

GLTPDQVVAIASNIGGKQALETVQRLLPVLCQDH

GLTPDQVVAIASHDGGKQALETVQRLLAVLCQDH

GLTPDQVVAIASHDGGKQALETVQRLLAVLCQDH

GLTPDQVVAIASNGGGKQALETVQRLLAVLCQDH

GLTPDQVVAIASNGGGKQALETVQRLLAVLCQDH

GLTPDQVVAIASNGGGKQALETVQRLLPVLCQDH

GLTPDQVVAIASNNGGKQALETVQRLLPVLCQDH

GLTPDQVVAIASNNGGKQALETVQRLLPVLCQDH

GLTPDQVVAIASNIGGKQALETVQRLLPVLCQDH

GLTPDQVVAIASNGGGKQALETVQRLLPVLCQDH

GLTPDQVVAIASNNGGKQALETVQRLLPVLCQDH

GLTPDQVVAIASNNGGKQALETVQRLLPVLCQDH

GLTPDQVVAIASNGGGKQALETVQRLLPVLCQDH

GLTPDQVVAIASNNGGKQALESIVAQLSRPDPALAALTNDHLVALACLGGRPAMDAVKKGLPHAPELIRRVNRRIGERTSHRVADYAQVVRVLEFFQCHSHPAYAFDEAMTQFGMSGQAGQASPKKKRKVGRADALDDFDLDMLGSDALDDFDLDMLGSDALDDFDLDMLGSDALDDFDLDMLINYPYDVPDYAS

**>TLT3-TF**

MAQAASGSPRPPRAKPAPRRRAAQPSDASPAAQVDLRTLGYSQQQQEKIKPKVRSTVAQHHEALVGHGFTHAHIVALSQHPAALGTVAVTYQHIITALPEATHEDIVGVGKQWSGARALEALLTDAGELRGPPLQLDTGQLVKIAKRGGVTAMEAVHASRNALTGAP

LNLTPDQVVAIASNGGGKQALETVQRLLPVLCQDH

GLTPDQVVAIASNNGGKQALETVQRLLPVLCQDH

GLTPDQVVAIASNGGGKQALETVQRLLAVLCQDH

GLTPDQVVAIASNGGGKQALETVQRLLAVLCQDH

GLTPDQVVAIASNIGGKQALETVQRLLAVLCQDH

GLTPDQVVAIASHDGGKQALETVQRLLAVLCQDH

GLTPDQVVAIASNIGGKQALETVQRLLPVLCQDH

GLTPDQVVAIASHDGGKQALETVQRLLPVLCQDH

GLTPDQVVAIASHDGGKQALETVQRLLPVLCQDH

GLTPDQVVAIASHDGGKQALETVQRLLPVLCQDH

GLTPDQVVAIASNGGGKQALETVQRLLPVLCQDH

GLTPDQVVAIASNNGGKQALETVQRLLPVLCQDH

GLTPDQVVAIASNGGGKQALETVQRLLPVLCQDH

GLTPDQVVAIASNNGGKQALETVQRLLPVLCQDH

GLTPDQVVAIASNIGGKQALESIVAQLSRPDPALAALTNDHLVALACLGGRPAMDAVKKGLPHAPELIRRVNRRIGERTSHRVADYAQVVRVLEFFQCHSHPAYAFDEAMTQFGMSGQAGQASPKKKRKVGRADALDDFDLDMLGSDALDDFDLDMLGSDALDDFDLDMLGSDALDDFDLDMLINYPYDVPDYAS

**>TLT4-TF**

MAQAASGSPRPPRAKPAPRRRAAQPSDASPAAQVDLRTLGYSQQQQEKIKPKVRSTVAQHHEALVGHGFTHAHIVALSQHPAALGTVAVTYQHIITALPEATHEDIVGVGKQWSGARALEALLTDAGELRGPPLQLDTGQLVKIAKRGGVTAMEAVHASRNALTGAP

LNLTPDQVVAIASHDGGKQALETVQRLLPVLCQDH

GLTPDQVVAIASNIGGKQALETVQRLLPVLCQDH

GLTPDQVVAIASHDGGKQALETVQRLLAVLCQDH

GLTPDQVVAIASNIGGKQALETVQRLLAVLCQDH

GLTPDQVVAIASNGGGKQALETVQRLLAVLCQDH

GLTPDQVVAIASNNGGKQALETVQRLLAVLCQDH

GLTPDQVVAIASNNGGKQALETVQRLLPVLCQDH

GLTPDQVVAIASHDGGKQALETVQRLLPVLCQDH

GLTPDQVVAIASHDGGKQALETVQRLLPVLCQDH

GLTPDQVVAIASHDGGKQALETVQRLLPVLCQDH

GLTPDQVVAIASNNGGKQALETVQRLLPVLCQDH

GLTPDQVVAIASNIGGKQALETVQRLLPVLCQDH

GLTPDQVVAIASNNGGKQALETVQRLLPVLCQDH

GLTPDQVVAIASNIGGKQALETVQRLLPVLCQDH

GLTPDQVVAIASNNGGKQALESIVAQLSRPDPALAALTNDHLVALACLGGRPAMDAVKKGLPHAPELIRRVNRRIGERTSHRVADYAQVVRVLEFFQCHSHPAYAFDEAMTQFGMSGQAGQASPKKKRKVGRADALDDFDLDMLGSDALDDFDLDMLGSDALDDFDLDMLGSDALDDFDLDMLINYPYDVPDYAS

**>TLT5-TF**

MAQAASGSPRPPRAKPAPRRRAAQPSDASPAAQVDLRTLGYSQQQQEKIKPKVRSTVAQHHEALVGHGFTHAHIVALSQHPAALGTVAVTYQHIITALPEATHEDIVGVGKQWSGARALEALLTDAGELRGPPLQLDTGQLVKIAKRGGVTAMEAVHASRNALTGAP

LNLTPDQVVAIASNNGGKQALETVQRLLPVLCQDH

GLTPDQVVAIASNNGGKQALETVQRLLPVLCQDH

GLTPDQVVAIASHDGGKQALETVQRLLAVLCQDH

GLTPDQVVAIASHDGGKQALETVQRLLAVLCQDH

GLTPDQVVAIASHDGGKQALETVQRLLAVLCQDH

GLTPDQVVAIASNNGGKQALETVQRLLAVLCQDH

GLTPDQVVAIASNIGGKQALETVQRLLPVLCQDH

GLTPDQVVAIASNNGGKQALETVQRLLPVLCQDH

GLTPDQVVAIASNIGGKQALETVQRLLPVLCQDH

GLTPDQVVAIASNNGGKQALETVQRLLPVLCQDH

GLTPDQVVAIASHDGGKQALETVQRLLPVLCQDH

GLTPDQVVAIASNGGGKQALETVQRLLPVLCQDH

GLTPDQVVAIASNNGGKQALETVQRLLPVLCQDH

GLTPDQVVAIASHDGGKQALETVQRLLPVLCQDH

GLTPDQVVAIASNIGGKQALESIVAQLSRPDPALAALTNDHLVALACLGGRPAMDAVKKGLPHAPELIRRVNRRIGERTSHRVADYAQVVRVLEFFQCHSHPAYAFDEAMTQFGMSGQAGQASPKKKRKVGRADALDDFDLDMLGSDALDDFDLDMLGSDALDDFDLDMLGSDALDDFDLDMLINYPYDVPDYAS

**>TLT6-TF**

MAQAASGSPRPPRAKPAPRRRAAQPSDASPAAQVDLRTLGYSQQQQEKIKPKVRSTVAQHHEALVGHGFTHAHIVALSQHPAALGTVAVTYQHIITALPEATHEDIVGVGKQWSGARALEALLTDAGELRGPPLQLDTGQLVKIAKRGGVTAMEAVHASRNALTGAP

LNLTPDQVVAIASNNGGKQALETVQRLLPVLCQDH

GLTPDQVVAIASHDGGKQALETVQRLLPVLCQDH

GLTPDQVVAIASNIGGKQALETVQRLLAVLCQDH

GLTPDQVVAIASNGGGKQALETVQRLLAVLCQDH

GLTPDQVVAIASHDGGKQALETVQRLLAVLCQDH

GLTPDQVVAIASHDGGKQALETVQRLLAVLCQDH

GLTPDQVVAIASNNGGKQALETVQRLLPVLCQDH

GLTPDQVVAIASNNGGKQALETVQRLLPVLCQDH

GLTPDQVVAIASNIGGKQALETVQRLLPVLCQDH

GLTPDQVVAIASNNGGKQALETVQRLLPVLCQDH

GLTPDQVVAIASNGGGKQALETVQRLLPVLCQDH

GLTPDQVVAIASNIGGKQALETVQRLLPVLCQDH

GLTPDQVVAIASHDGGKQALETVQRLLPVLCQDH

GLTPDQVVAIASNGGGKQALETVQRLLPVLCQDH

GLTPDQVVAIASNIGGKQALESIVAQLSRPDPALAALTNDHLVALACLGGRPAMDAVKKGLPHAPELIRRVNRRIGERTSHRVADYAQVVRVLEFFQCHSHPAYAFDEAMTQFGMSGQAGQASPKKKRKVGRADALDDFDLDMLGSDALDDFDLDMLGSDALDDFDLDMLGSDALDDFDLDMLINYPYDVPDYAS

**>TLT7-TF**

MAQAASGSPRPPRAKPAPRRRAAQPSDASPAAQVDLRTLGYSQQQQEKIKPKVRSTVAQHHEALVGHGFTHAHIVALSQHPAALGTVAVTYQHIITALPEATHEDIVGVGKQWSGARALEALLTDAGELRGPPLQLDTGQLVKIAKRGGVTAMEAVHASRNALTGAP

LNLTPDQVVAIASNNGGKQALETVQRLLPVLCQDH

GLTPDQVVAIASHDGGKQALETVQRLLPVLCQDH

GLTPDQVVAIASNGGGKQALETVQRLLAVLCQDH

GLTPDQVVAIASNNGGKQALETVQRLLAVLCQDH

GLTPDQVVAIASNIGGKQALETVQRLLAVLCQDH

GLTPDQVVAIASHDGGKQALETVQRLLAVLCQDH

GLTPDQVVAIASNIGGKQALETVQRLLPVLCQDH

GLTPDQVVAIASNGGGKQALETVQRLLPVLCQDH

GLTPDQVVAIASHDGGKQALETVQRLLPVLCQDH

GLTPDQVVAIASNNGGKQALETVQRLLPVLCQDH

GLTPDQVVAIASNIGGKQALETVQRLLPVLCQDH

GLTPDQVVAIASNNGGKQALETVQRLLPVLCQDH

GLTPDQVVAIASHDGGKQALETVQRLLPVLCQDH

GLTPDQVVAIASNGGGKQALETVQRLLPVLCQDH

GLTPDQVVAIASNGGGKQALESIVAQLSRPDPALAALTNDHLVALACLGGRPAMDAVKKGLPHAPELIRRVNRRIGERTSHRVADYAQVVRVLEFFQCHSHPAYAFDEAMTQFGMSGQAGQASPKKKRKVGRADALDDFDLDMLGSDALDDFDLDMLGSDALDDFDLDMLGSDALDDFDLDMLINYPYDVPDYAS

**>TLT8-TF**

MAQAASGSPRPPRAKPAPRRRAAQPSDASPAAQVDLRTLGYSQQQQEKIKPKVRSTVAQHHEALVGHGFTHAHIVALSQHPAALGTVAVTYQHIITALPEATHEDIVGVGKQWSGARALEALLTDAGELRGPPLQLDTGQLVKIAKRGGVTAMEAVHASRNALTGAP

LNLTPDQVVAIASNGGGKQALETVQRLLPVLCQDH

GLTPDQVVAIASNGGGKQALETVQRLLPVLCQDH

GLTPDQVVAIASHDGGKQALETVQRLLAVLCQDH

GLTPDQVVAIASHDGGKQALETVQRLLAVLCQDH

GLTPDQVVAIASNNGGKQALETVQRLLAVLCQDH

GLTPDQVVAIASHDGGKQALETVQRLLAVLCQDH

GLTPDQVVAIASNGGGKQALETVQRLLPVLCQDH

GLTPDQVVAIASNNGGKQALETVQRLLPVLCQDH

GLTPDQVVAIASNNGGKQALETVQRLLPVLCQDH

GLTPDQVVAIASNNGGKQALETVQRLLPVLCQDH

GLTPDQVVAIASNNGGKQALETVQRLLPVLCQDH

GLTPDQVVAIASNIGGKQALETVQRLLPVLCQDH

GLTPDQVVAIASHDGGKQALETVQRLLPVLCQDH

GLTPDQVVAIASNGGGKQALETVQRLLPVLCQDH

GLTPDQVVAIASNGGGKQALESIVAQLSRPDPALAALTNDHLVALACLGGRPAMDAVKKGLPHAPELIRRVNRRIGERTSHRVADYAQVVRVLEFFQCHSHPAYAFDEAMTQFGMSGQAGQASPKKKRKVGRADALDDFDLDMLGSDALDDFDLDMLGSDALDDFDLDMLGSDALDDFDLDMLINYPYDVPDYAS

**>TLT9-TF**

MAQAASGSPRPPRAKPAPRRRAAQPSDASPAAQVDLRTLGYSQQQQEKIKPKVRSTVAQHHEALVGHGFTHAHIVALSQHPAALGTVAVTYQHIITALPEATHEDIVGVGKQWSGARALEALLTDAGELRGPPLQLDTGQLVKIAKRGGVTAMEAVHASRNALTGAP

LNLTPDQVVAIASNNGGKQALETVQRLLPVLCQDH

GLTPDQVVAIASNNGGKQALETVQRLLPVLCQDH

GLTPDQVVAIASHDGGKQALETVQRLLAVLCQDH

GLTPDQVVAIASNNGGKQALETVQRLLAVLCQDH

GLTPDQVVAIASNIGGKQALETVQRLLAVLCQDH

GLTPDQVVAIASNNGGKQALETVQRLLAVLCQDH

GLTPDQVVAIASHDGGKQALETVQRLLPVLCQDH

GLTPDQVVAIASHDGGKQALETVQRLLPVLCQDH

GLTPDQVVAIASHDGGKQALETVQRLLPVLCQDH

GLTPDQVVAIASNGGGKQALETVQRLLPVLCQDH

GLTPDQVVAIASHDGGKQALETVQRLLPVLCQDH

GLTPDQVVAIASNIGGKQALETVQRLLPVLCQDH

GLTPDQVVAIASNNGGKQALETVQRLLPVLCQDH

GLTPDQVVAIASNIGGKQALETVQRLLPVLCQDH

GLTPDQVVAIASNGGGKQALESIVAQLSRPDPALAALTNDHLVALACLGGRPAMDAVKKGLPHAPELIRRVNRRIGERTSHRVADYAQVVRVLEFFQCHSHPAYAFDEAMTQFGMSGQAGQASPKKKRKVGRADALDDFDLDMLGSDALDDFDLDMLGSDALDDFDLDMLGSDALDDFDLDMLINYPYDVPDYAS

**>TLT10-TF**

MAQAASGSPRPPRAKPAPRRRAAQPSDASPAAQVDLRTLGYSQQQQEKIKPKVRSTVAQHHEALVGHGFTHAHIVALSQHPAALGTVAVTYQHIITALPEATHEDIVGVGKQWSGARALEALLTDAGELRGPPLQLDTGQLVKIAKRGGVTAMEAVHASRNALTGAP

LNLTPDQVVAIASNGGGKQALETVQRLLPVLCQDH

GLTPDQVVAIASNIGGKQALETVQRLLPVLCQDH

GLTPDQVVAIASNGGGKQALETVQRLLAVLCQDH

GLTPDQVVAIASNGGGKQALETVQRLLAVLCQDH

GLTPDQVVAIASNNGGKQALETVQRLLAVLCQDH

GLTPDQVVAIASNIGGKQALETVQRLLAVLCQDH

GLTPDQVVAIASNNGGKQALETVQRLLPVLCQDH

GLTPDQVVAIASNNGGKQALETVQRLLPVLCQDH

GLTPDQVVAIASHDGGKQALETVQRLLPVLCQDH

GLTPDQVVAIASNGGGKQALETVQRLLPVLCQDH

GLTPDQVVAIASNGGGKQALETVQRLLPVLCQDH

GLTPDQVVAIASNIGGKQALETVQRLLPVLCQDH

GLTPDQVVAIASNIGGKQALETVQRLLPVLCQDH

GLTPDQVVAIASNNGGKQALETVQRLLPVLCQDH

GLTPDQVVAIASHDGGKQALESIVAQLSRPDPALAALTNDHLVALACLGGRPAMDAVKKGLPHAPELIRRVNRRIGERTSHRVADYAQVVRVLEFFQCHSHPAYAFDEAMTQFGMSGQAGQASPKKKRKVGRADALDDFDLDMLGSDALDDFDLDMLGSDALDDFDLDMLGSDALDDFDLDMLINYPYDVPDYAS

**S1 Table. TALE proteins sequences used in this study.** TALE N-terminal domain is colored orange. TALE DNA-binding domain is colored blue. RVD residues are shown in red. Nuclear localization signal (NLS) sequence is highlighted grey. VP64 domain is colored green. HA tag is colored purple.
