# Supplementary material for: Reactivation of Latent HIV-1 Expression by Engineered TALE Transcription Factors
Source: PLoS One. 2016 Mar 2;11(3):e0150037. doi: 10.1371/journal.pone.0150037 (PMC4774903; doi:10.1371/journal.pone.0150037)
Supplement: S2 Table — TALE binding sites are underlined. Restriction sites are in bold. (DOCX) [file pone.0150037.s005.docx]

**>5’ TALE-Luc-TLT1**

ACTGCTAT**CTCGAG**TACCACACACAAGGCTTAGCGTACCACACACAAGGCTTAGCGTACCACACACAAGGCTTAGCGTACCACACACAAGGCTTAGCGATCTGCGATCTAAGTAAGCT

**>5’ TALE-Luc-TLT2**

ACTGCTAT**CTCGAG**TGACCTTTGGATGGTGTAGCGTGACCTTTGGATGGTGTAGCGTGACCTTTGGATGGTGTAGCGTGACCTTTGGATGGTGTAGCGATCTGCGATCTAAGTAAGCT

**>5’ TALE-Luc-TLT3**

ACTGCTAT**CTCGAG**TTGTTACACCCTGTGATAGCGTTGTTACACCCTGTGATAGCGTTGTTACACCCTGTGATAGCGTTGTTACACCCTGTGATAGCGATCTGCGATCTAAGTAAGCT

**>5’ TALE-Luc-TLT4**

ACTGCTAT**CTCGAG**TCACATGGCCCGAGAGTAGCGTCACATGGCCCGAGAGTAGCGTCACATGGCCCGAGAGTAGCGTCACATGGCCCGAGAGTAGCGATCTGCGATCTAAGTAAGCT

**>5’ TALE-Luc-TLT5**

ACTGCTAT**CTCGAG**TGGCCCGAGAGCTGCATAGCGTGGCCCGAGAGCTGCATAGCGTGGCCCGAGAGCTGCATAGCGTGGCCCGAGAGCTGCATAGCGATCTGCGATCTAAGTAAGCT

**>5’ TALE-Luc-TLT6**

ACTGCTAT**CTCGAG**TGCATCCGGAGTACTATAGCGTGCATCCGGAGTACTATAGCGTGCATCCGGAGTACTATAGCGTGCATCCGGAGTACTATAGCGATCTGCGATCTAAGTAAGCT

**>5’ TALE-Luc-TLT7**

ACTGCTAT**CTCGAG**TGCTGACATCGAGCTTTAGCGTGCTGACATCGAGCTTTAGCGTGCTGACATCGAGCTTTAGCGTGCTGACATCGAGCTTTAGCGATCTGCGATCTAAGTAAGCT

**>5’ TALE-Luc-TLT8**

ACTGCTAT**CTCGAG**TTTCCGCTGGGGACTTTAGCGTTTCCGCTGGGGACTTTAGCGTTTCCGCTGGGGACTTTAGCGTTTCCGCTGGGGACTTTAGCGATCTGCGATCTAAGTAAGCT

**>5’ TALE-Luc-TLT9**

ACTGCTAT**CTCGAG**TGGCGAGCCCTCAGATTAGCGTGGCGAGCCCTCAGATTAGCGTGGCGAGCCCTCAGATTAGCGTGGCGAGCCCTCAGATTAGCGATCTGCGATCTAAGTAAGCT

**>5’ TALE-Luc-TLT10**

ACTGCTAT**CTCGAG**TTATTGAGGCTTAAGCTAGCGTTATTGAGGCTTAAGCTAGCGTTATTGAGGCTTAAGCTAGCGTTATTGAGGCTTAAGCTAGCGATCTGCGATCTAAGTAAGCT

**>3’ Luc-Rev**

CGTTTTCCCGGTACCAGAT

**S2 Table. Primer sequences for the construction of the luciferase reporter plasmids used in this study.** TALE binding sites are underlined. Restriction sites are in bold.
