# Supplementary material for: Reactivation of Latent HIV-1 Expression by Engineered TALE Transcription Factors
Source: PLoS One. 2016 Mar 2;11(3):e0150037. doi: 10.1371/journal.pone.0150037 (PMC4774903; doi:10.1371/journal.pone.0150037)
Supplement: S3 Table — Data based on 2014 edition of the HIV Sequence Database (http://hiv-web.lanl.gov). Dashes indicate sequence identity between subtype strains. Dots indicate gaps in the HIV genome sequence. (DOCX) [file pone.0150037.s006.docx]

|  | **TLT5** | **TLT6** | **TLT7** | **TLT8** |
| --- | --- | --- | --- | --- |
| **HIV strain** | TGGCCCGAGAGCTGCA | TGCATCCGGAGTACTA | TGCTGACATCGAGCTT | TTTCCGCTGGGGACTT |
| **B.FR.83.HXB2_LAI_IIIB_BRU.K03455** | ---------------- | ---------------T | ---------------- | ---------------- |
| **B.AU.86.MBC200.AF042100** | ---------------- | ---------------- | --A----C-------- | ---------------- |
| **B.AU.87.MBC925.AF042101** | ---------------- | ---------------- | --------G------- | ---------------- |
| **B.AU.95.C24.AF538304** | -----AA--------- | ---------------- | ---------T------ | ---------------- |
| **B.BR.02.02BR011.DQ358809** | -----------AAA-- | AA-------------- | --------G----T-- | ---------------- |
| **B.CN.02.02HNsc11.DQ007903** | -----A---------- | ---------------- | -------------T-- | ------T--------- |
| **B.CN.05.05CNHB_hp3.DQ990880** | -----------AAA-- | AA-----T----T--- | --------CT-----A | ---------------- |
| **B.ES.89.U61.DQ854716** | A------------A-- | -A-------------- | ---------------- | ---------------- |
| **B.GB.83.CAM1.D10112** | -----------AA--- | A-----------T--- | ---------------- | ---------------- |
| **B.GB.86.GB8_46R.AJ271445** | -------C--A-C--- | C-----------TT-- | -----------GAG-- | ---------------- |
| **B.GB.x.MANC.U23487** | ---------------- | ---------------- | --------GGC----- | --------A------- |
| **B.JP.00.DR2508.AB289588** | -----A---------- | ----------T----- | -----------.AG-- | ---------------- |
| **B.JP.04.DR5913.AB480696** | -------C-----A-- | -A----------TT-- | ---------------- | ---------------- |
| **B.JP.05.DR6538.AB287363** | A-----------AA-- | AA----------TT-- | -------------T-- | ---------------- |
| **B.JP.98.DR1120.AB480698** | -----------AAA-- | AA----------TT-- | -------------A-- | ---------------- |
| **B.KR.03.03KGS5.JQ316132** | -----------AAA-- | AA-------------- | -------------AAC | ---------------- |
| **B.KR.04.04LHS6.AY839827** | .....----------- | ---------------- | --------C------- | ---------------- |
| **B.KR.05.05YJN2.JQ316134** | -A---------AAA-- | AA-------------- | --------CT------ | ---------------- |
| **B.NL.96.H434_42_A1.AY970948** | ---------------- | ---------------- | ---------------- | ---------------- |
| **B.TW.94.TWCYS_LM49.AF086817** | GA-----------A-- | -A-------------- | --------Y------- | -------.T------- |
| **B.US.00.ES1_20.EF363123** | ---------------- | ---------------T | ---------------- | ---------------- |
| **B.US.01.REJO_TF1.JN944911** | -----------AA--- | A--------------T | --------C------- | --------.------- |
| **B.US.04.ES4_24.EF363124** | ---------------- | ---------------- | ---------------- | ---------------- |
| **B.US.06.CH106_TF1.JN944897** | -A---------AAA-- | AA----------T--- | ---------------- | ---------------- |
| **B.US.09.C1P.GU733713** | -A---------AAA-- | AA-------------T | --------C--CT--C | ---------------- |
| **B.US.10.VC1.JN397364** | -A---------AAA-- | AA-------------- | ---------------- | ---------------- |
| **B.US.11.CP10_3A.KF384798** | ---------------- | ----------T----- | --------G------- | ---------------- |
| **B.US.83.5018_83.AY835777** | ---------------- | ---------------- | ---------------- | ---------------- |
| **B.US.84.5019_84.AY835779** | ---------------- | ---------------- | ---------------- | ---------------- |
| **B.US.85.5077_85.AY835769** | ---------------- | ---------------- | ---------------- | ---------------- |
| **B.US.86.5084_86.AY835775** | ---------------- | ---------------- | ---------------- | ---------------- |
| **B.US.87.5113_87.AY835758** | ---------------- | ---------------- | ---------------- | ---------------- |
| **B.US.88.5160_88.AY835763** | ---------------- | ---------------- | ---------------- | ---------------- |
| **B.US.89.P896_89_6.U39362** | ---------------- | ---------------T | --------CT-----A | ---------------- |
| **B.US.90.WEAU160_GHOSH.U21135** | -A-------------- | ---------------T | -------------T-- | -----A---------- |
| **B.US.91.5048_91.AY835761** | ---------------- | ---------------- | ---------------- | ---------------- |
| **B.US.94.5082_94.AY835773** | ---------------- | ---------------- | ---------------- | ---------------- |
| **B.US.95.5073_95.AY835768** | ---------------- | ---------------- | ---------------- | ---------------- |

**S3 Table. Sequence conservation of the TALE transcription factor binding sites across HIV-1 subtype B strains.** Data based on 2014 edition of the HIV Sequence Database (http://hiv-web.lanl.gov). Dashes indicate sequence identity between subtype strains. Dots indicate gaps in the HIV genome sequence.
